# Friction forces determine cytoplasmic reorganization and shape changes of ascidian oocytes upon fertilization

**DOI:** 10.1038/s41567-023-02302-1

**Published:** 2024-01-09

**Authors:** Silvia Caballero-Mancebo, Rushikesh Shinde, Madison Bolger-Munro, Matilda Peruzzo, Gregory Szep, Irene Steccari, David Labrousse-Arias, Vanessa Zheden, Jack Merrin, Andrew Callan-Jones, Raphaël Voituriez, Carl-Philipp Heisenberg

**Affiliations:** 1 Institute of Science and Technology Austria, Klosterneuburg, Austria; 2 Laboratoire de Matière et Systèmes Complexes, Université de Paris Cité and CNRS, Paris, France; 3 King’s College London, London, UK; 4 Laboratoire Jean Perrin, Sorbonne Université and CNRS, Paris, France; 5 Laboratoire de Physique Théorique de la Matière Condensée, Sorbonne Université and CNRS, Paris, France

**Keywords:** Biophysics, Biological physics

## Abstract

Contraction and flow of the actin cell cortex have emerged as a common principle by which cells reorganize their cytoplasm and take shape. However, how these cortical flows interact with adjacent cytoplasmic components, changing their form and localization, and how this affects cytoplasmic organization and cell shape remains unclear. Here we show that in ascidian oocytes, the cooperative activities of cortical actomyosin flows and deformation of the adjacent mitochondria-rich myoplasm drive oocyte cytoplasmic reorganization and shape changes following fertilization. We show that vegetal-directed cortical actomyosin flows, established upon oocyte fertilization, lead to both the accumulation of cortical actin at the vegetal pole of the zygote and compression and local buckling of the adjacent elastic solid-like myoplasm layer due to friction forces generated at their interface. Once cortical flows have ceased, the multiple myoplasm buckles resolve into one larger buckle, which again drives the formation of the contraction pole—a protuberance of the zygote’s vegetal pole where maternal mRNAs accumulate. Thus, our findings reveal a mechanism where cortical actomyosin network flows determine cytoplasmic reorganization and cell shape by deforming adjacent cytoplasmic components through friction forces.

## Abstract (web summary)

Friction forces at the interface between tissues play a key role in tissue morphogenesis. Now friction at the cellular scale is shown to influence cell shape and cell rearrangements.

## Main

Cytoplasmic reorganization is an essential feature of many core cell biological processes, such as cell division and cell migration^1–3^. In oocytes, cytoplasmic (ooplasmic) reorganization plays a key role in redistributing different organelles, such as the nucleus and meiotic spindle^4,5^, and maternal determinants^6^, thereby laying down the blueprint for subsequent embryo patterning and morphogenesis^7^.

Several studies have highlighted the importance of the cytoskeleton, especially the cortical actomyosin network, in mediating cytoplasmic reorganization^7^. In the *Caenorhabditis elegans* zygote, for instance, cortical actomyosin flows directed towards the anterior pole are triggered as a result of myosin II downregulation at the posterior pole. These flows further polarize the cortex of the zygote and transport proteins implicated in anterior–posterior axis specification^8–11^. Similarly, periodic cortical actomyosin contraction waves in the starfish oocyte lead to changes in oocyte shape and the establishment of ooplasmic flows^12,13^.

Friction forces arising at the interface between adjacent embryonic tissues have recently been suggested to play important roles in embryo morphogenesis. In *Tribolium* and *Drosophila* embryos, blastoderm tissue movements during gastrulation are resisted by friction forces with the adjacent vitelline envelope, a rigid shell-like structure surrounding the embryo, leading to asymmetric tissue flows important for proper tissue positioning and embryo development^14,15^. Likewise, in zebrafish embryos, friction forces arising at the interface between the mesendodermal and ectodermal germ layers play a decisive role in neuroectoderm morphogenesis^16^. These findings indicate that at the tissue scale, friction arising at the boundary between differentially moving tissues constitutes an important force-generating process driving embryo morphogenesis.

In ascidian oocytes, contraction of the oocyte cortex along its animal–vegetal (AV) axis upon fertilization has been linked to the relocalization and eventual accumulation of cortical endoplasmic reticulum (cER) and associated maternal mRNAs in a region at the vegetal pole (VP) called the ‘contraction pole’ (CP), a process essential for subsequent establishment of the embryonic axes^17,18^. The formation of the CP becomes morphologically detectable as a transient protuberance at the VP of the zygote, but the mechanisms underlying CP formation and the potential function of cortical actomyosin contraction therein are unknown.

## Shape changes of the ascidian oocyte upon fertilization

**Fig. 1:**
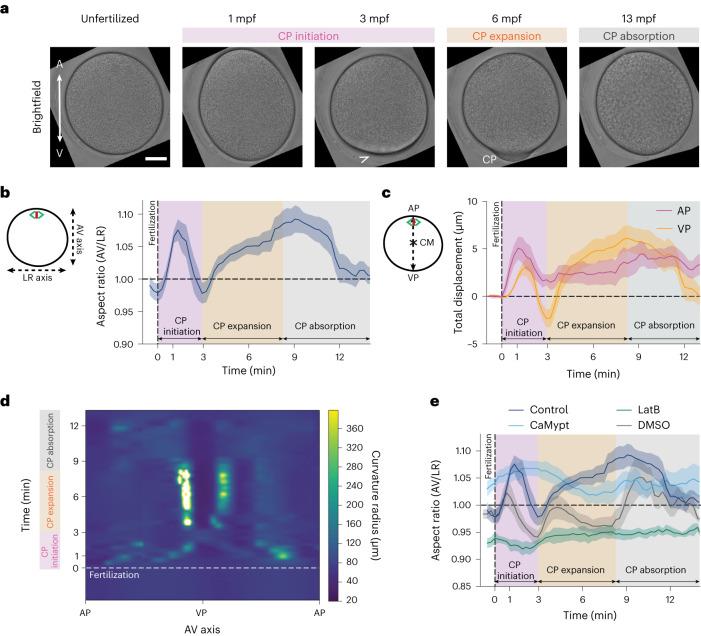
Oocytes undergo pronounced cell shape changes following fertilization. **a**, Brightfield confocal cross-sections of a representative oocyte/zygote during CP morphogenesis. Arrowhead points at the vegetal bulge formed at the end of CP initiation. Scale bar, 30 µm. **b**,**c**, Plot of oocyte AR (**b**) and the distance from the AP and VP to the CM (**c**) as a function of time after fertilization (0 min, dashed vertical line). The dotted line at 0 min indicates the fertilization time point. The pink-shaded area corresponds to CP initiation, the orange-shaded area to CP expansion, and the grey-shaded area to CP absorption. *N* = 8 animals, *n* = 10 oocytes. Error bars, standard error of the mean (s.e.m.). **d**, Exemplary kymograph of the curvature radius around the oocyte perimeter shown in **a** as a function of time after fertilization (0 min, dashed horizontal line). Colour bar indicates the curvature radius in µm. **e**, Quantification of oocyte AR as a function of time after fertilization (0 min, dashed vertical line) in control (**a**), dimethyl sulfoxide (DMSO, 0.1%, *N* = 3 animals, *n* = 5 oocytes), LatB-treated (1 µg ml^−1^, *N* = 4 animals, *n* = 5 oocytes) and CaMypt-overexpressing oocytes (*N* = 3 animals, *n* = 5 oocytes). Error bars, s.e.m. A, AP; V, VP; LR, left–right. Source data

The unfertilized oocyte of the ascidian *Phallusia mammillata* undergoes a series of cellular deformations upon fertilization, culminating in the formation of the CP, a distinct protuberance at the VP of the zygote where cER and associated maternal mRNAs, which are needed for subsequent embryo patterning and morphogenesis, accumulate^18^ (Fig. 1a and Supplementary Video 1). To elucidate the mechanisms leading to CP formation, we first analysed changes in the overall shape of the oocyte during CP morphogenesis by measuring the aspect ratio (AR) of the oocyte (Fig. 1b), the absolute displacement of both the animal pole (AP) and VP from the centre of mass (CM) (Fig. 1c), and the changes in the radius of curvature along the perimeter of the oocyte (Fig. 1d) during this process. This analysis revealed three distinct phases of CP morphogenesis. In the first phase (‘CP initiation’), the animal and vegetal poles expand along the AV axis upon fertilization, leading to an increase in the curvature radius between the AP and VP (Fig. 1d, pink area) and giving the zygote an overall ellipsoid shape (Fig. 1a, 1 minute postfertilization, (mpf)), followed by a retraction of the VP and the formation of a small vegetal bulge (Fig. 1a, arrowhead, and Fig. 1b,c, pink area). In the second phase (‘CP expansion’), a much larger local protuberance forms at the VP, giving the CP its characteristic bell shape (Fig. 1b–d, orange area). In the third and final phase (‘CP absorption’), the CP slowly retracts (Fig. 1b–d, grey area). The entire process of CP morphogenesis lasts 14 minutes, with CP initiation taking ~3 mins, CP expansion ~6 mins and CP absorption ~5 mins.

## Cortical actomyosin flows towards the VP during CP initiation

**Extended Data Fig. 1:**
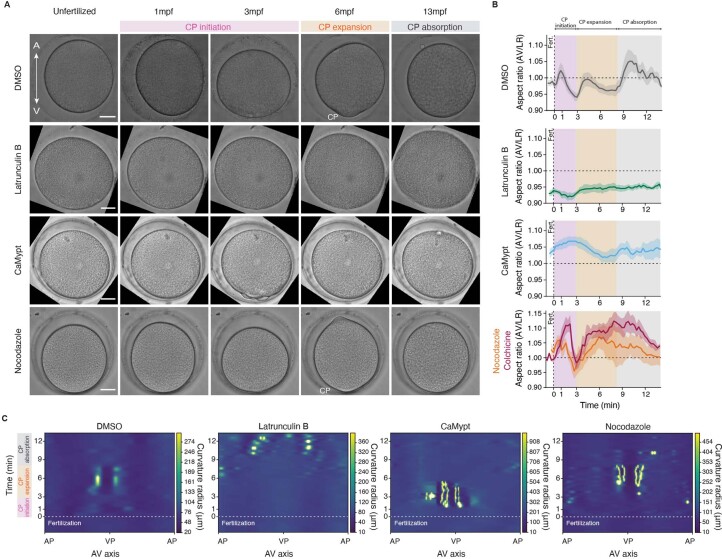
Changes in the oocyte cell shape depend on the activity of the actomyosin cortex. **A**, Brightfield confocal cross-sections of a representative oocyte/zygote during CP morphogenesis after treatment with DMSO (0.1%), LatB (1 µg ml^−1^) and Nocodazole (0.6 µM), and overexpression of CaMypt. Scale bar, 30 µm. **B**, Plot of the oocyte aspect ratio as a function of time after fertilization (0 min, dashed vertical line) after treatment with DMSO (0.1%, *N* = 4 animals, *n* = 5 oocytes), LatB (1 µg ml^−1^, *N* = 4 animals, *n* = 5 oocytes), and Nocodazole (0.6 µM, orange line, *N* = 3 animals, *n* = 4 oocytes) and Colchicine (100 µM, red line, *N* = 3 animals, *n* = 6 oocytes), and overexpression of CaMypt (*N* = 3 animals, *n* = 5 oocytes). Error bars, s.e.m., **C**, Kymographs of the curvature radius around the oocyte perimeter as a function of time after fertilization (0 min, dashed horizontal line) for the treatments shown in (**A**). Color bars indicate the respective curvature radius in µm. Source data

**Extended Data Fig. 2:**
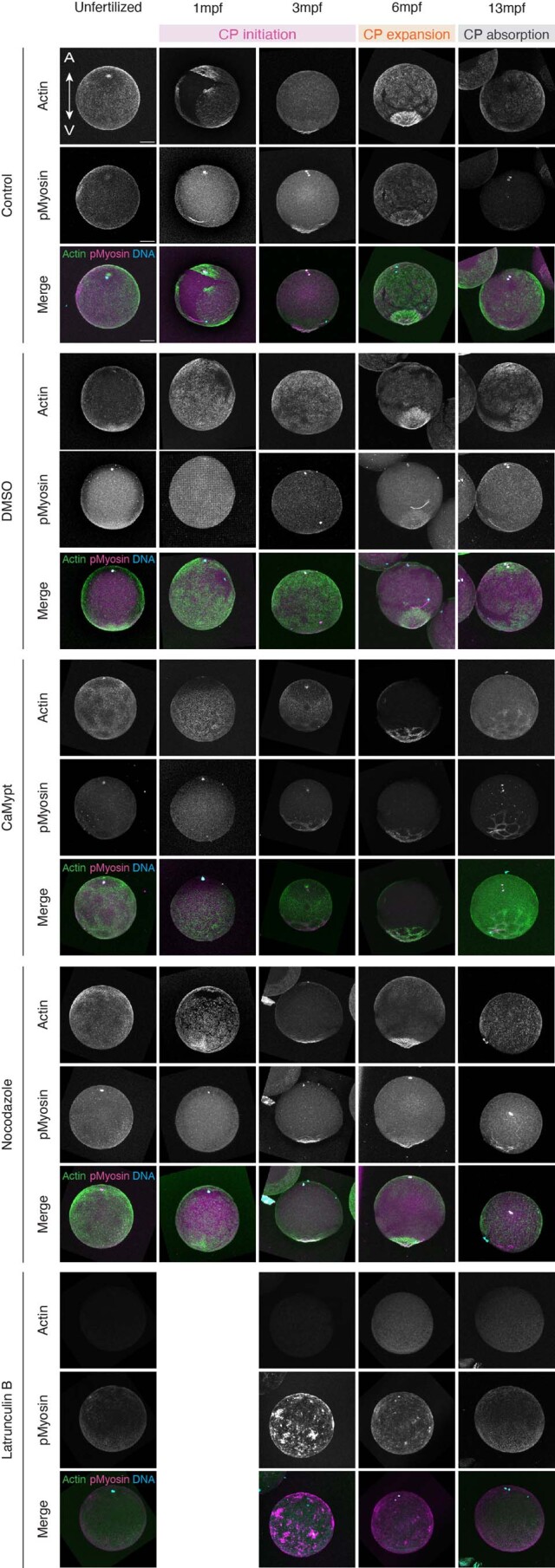
Interfering with the actomyosin, but not microtubule cytoskeleton, affects oocyte shape changes. Maximum intensity projections of F-actin (green) and pMyosin (magenta) in fixed control oocytes and oocytes at different stages of CP morphogenesis exposed to DMSO (0.1%), injected with CaMypt mRNA ( ~ 0.3 nl of 1.5 µg µl^−1^ mRNA, myosin II inhibition), and treated with Nocodazole (0.6 µM, microtubule disruption) or LatB (1 µg µl^−1^; F-actin disruption). F-actin was detected by Phalloidin and pMyosin by immunohistochemistry. Note that, due to the lack of deformations in LatB-treated oocytes upon fertilization, fertilized LatB-treated oocytes could only be reliably identified from 3mpf onwards. Scale bar, 30 µm. Source data

Next, we asked which biomechanical processes might drive CP formation. Previous studies have proposed that actomyosin-dependent cortical contraction, initiated upon oocyte fertilization, leads to CP formation^17,19^. To test this possibility, we interfered with actomyosin contraction by exposing oocytes to 1 µg ml^−1^ Latrunculin B (LatB), depolymerizing actin, and overexpressing a constitutive active variant of myosin-phosphatase (CaMypt), blocking myosin II activity^20^. Both treatments led to strongly diminished CP formation (Fig. 1e, Extended Data Fig. 1a–c and Supplementary Video 2), with CaMypt overexpression eliciting a generally milder phenotype than treatment with LatB, likely because of residual activity of myosin II in the overexpressing oocytes (Extended Data Fig. 1a,c, CaMypt). This supports the notion that actomyosin contraction is required for CP formation. By contrast, interfering with the microtubule cytoskeleton by exposing oocytes to 0.6 µM Nocodazole or 100 µM Colchicine had no specific effect on CP formation (Extended Data Fig. 1a–c, Nocodazole/Colchicine, and Supplementary Video 2), arguing against a critical function of microtubules in this process. Of note, the initial increase in the AR of the oocyte during CP initiation was less pronounced in Nocodazole-treated as compared to control embryos, likely because of Nocodazole affecting meiotic spindle formation and thus polar body extrusion at the AP. Treating oocytes with DMSO (control) also slightly affected oocyte shape; however, this effect was not observed in the presence of Nocodazole or Colchicine (Extended Data Fig. 1a–c). The effectiveness of these different drug treatments was further tested by immunohistochemistry, showing that the targeted cytoskeletal networks were severely affected in treated oocytes (Extended Data Fig. 2).

**Fig. 2:**
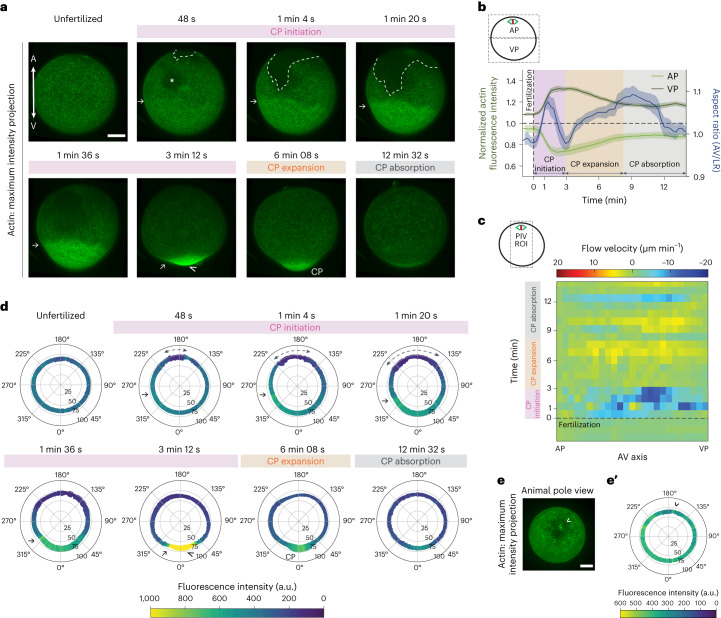
Cortical actin flows towards the VP during CP initiation. **a**, Confocal fluorescence images of maximum intensity projections of cortical actin (Utrophin-Venus) distribution in a representative oocyte during consecutive stages of CP morphogenesis. Asterisk demarcates the point of sperm entry. Arrows point at the transition zone between low- and high-intensity cortical actin domains. Arrowhead points at the vegetal bulge formed at the end of CP initiation. Dashed line demarcates the opening of the actin cortex at the AP upon fertilization. Scale bar, 30 µm. **b**, Plot of total actin intensity in the AP and VP hemispheres of oocytes/zygotes (*N* = 6 animals, *n* = 7 oocytes; green, left *y* axis) and their AR (taken from Fig. 1b, blue, right *y* axis) as a function of time after fertilization (0 min, dashed vertical line). Error bars, s.e.m. **c**, Kymograph of cortical actin flow velocity along the AV axis as a function of time after fertilization (0 min, dashed horizontal line). Colour bar indicates velocity in µm min^-1^ (positive velocities towards the AP, negative velocities towards the VP). **d**, Polar plots of sum intensity projections of cortical actin (Utrophin-Venus) over 10 central z-slides (15 µm). Angles represent the position around the oocyte (VP is at 0°, AP at 180°). The radius of the oocyte (in µm) is indicated in the concentric circles. Arrows point at the transition zone between low- and high-intensity cortical actin domains identified in **a**. Dashed arrows point at the opening of the actin cortex at the AP. Arrowhead points at the vegetal bulge formed at the end of CP initiation. Colour bar indicates fluorescence intensity in arbitrary units. **e**, Confocal fluorescence maximum intensity projection of cortical actin in an unfertilized oocyte. Arrowhead, patch of actin accumulation adjacent to the meiotic spindle at the AP. Scale bar, 30 µm. **e′**, Polar plot of sum intensity projections of cortical actin over 10 central z-slides of an unfertilized oocyte. Angles represent the position around the oocyte (VP, 0°; AP, 180°). Colour bar indicates fluorescence intensity in arbitrary units.

**Extended Data Fig. 3:**
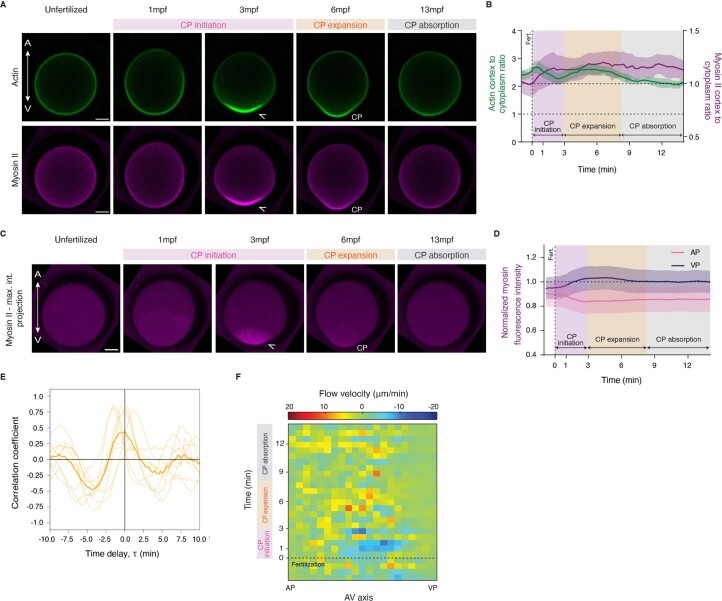
Cortical actomyosin dynamics during CP formation. **A**, Confocal fluorescence images of sum intensity projections over 10 central z-slides of cortical actin (top row) and cortical myosin II (bottom row) distribution in a representative oocyte during CP morphogenesis. Arrowheads,vegetal bulge. Scale bar, 30 µm. **B**, Plot of the ratio of cortical to cytoplasmic intensities for actin (left y-axis, *N* = 5 animals, *n* = 6 oocytes, green) and myosin II (right y-axis, *N* = 5 animals, *n* = 6 oocytes, magenta) as a function of time after fertilization (0 min, dashed vertical line). Error bars, s.e.m. **C** Confocal fluorescence images of maximum intensity projections of cortical myosin II distribution in a representative oocyte during CP morphogenesis. Arrowhead, vegetal bulge. Scale bar, 30 µm. **D**, Plot of the total myosin II intensity in the animal (AP) and vegetal (VP) hemispheres of oocytes/zygotes (*N* = 5 animals, *n* = 8 oocytes) as a function of time after fertilization (0 min, dashed vertical line). Error bars, s.e.m. **E**, Plot of temporal cross-correlation analysis between actin (*N* = 3 animals, *n* = 3 oocytes) and myosin II (*N* = 3 animals, *n* = 3 oocytes) total intensities. Individual actin-myosin II cross-correlations (semi-transparent orange lines) and the average (opaque orange line) are shown. **F**, Kymograph of myosin II flow velocity along the AV axis as a function of time after fertilization (0 min, dashed horizontal line). Color bar indicates velocity in µm min^−1^ (positive velocities towards the AP, negative velocities towards the VP). Source data

To identify the mechanisms by which actomyosin contraction functions in CP formation, we analysed dynamic changes in the subcellular localization of actin and myosin II during CP morphogenesis (Extended Data Fig. 3a and Supplementary Video 3). Consistent with previous observations^21^, this showed that before fertilization, both actin and myosin II predominantly localize to the oocyte cortex (Extended Data Fig. 3a,b), with slightly higher accumulation at the VP than the AP (Fig. 2a,b and Extended Data Fig. 3c,d, unfertilized), and that their localization was correlated in time (Extended Data Fig. 3e). Upon fertilization and during CP initiation, this unequal distribution of cortical actomyosin was rapidly and strongly enhanced, accompanied by the formation of a characteristic small bulge at the VP (Fig. 2a and Extended Data Fig. 3a,c, arrowhead). Unexpectedly, however, actomyosin distribution along the AV axis of the oocyte nearly equalized again during CP expansion (Fig. 2a,b and Extended Data Fig. 3c,d, orange area), pointing at the possibility that CP initiation and expansion are regulated differently by cortical actomyosin.

**Extended Data Fig. 4:**
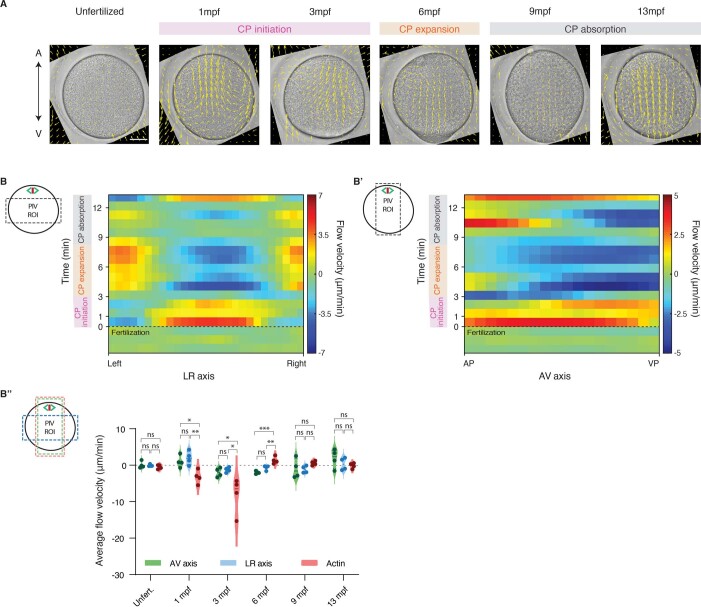
Bulk cytoplasmic flows during CP formation. **A**, Brightfield images (cross-sections) of a representative oocyte at consecutive timepoints during CP morphogenesis and PIV analysis of bulk cytoplasmic flows (yellow arrows). Scale bar, 30 µm. **B** and **B’**, Kymographs of bulk cytoplasmic flow velocities along the oocyte AV axis as a function of time after fertilization (0 min, dashed horizontal line) analyzed in a region perpendicular (**B**) and parallel (**B’**) to the oocyte AV axis. Colour bar indicates velocity in µm min^−1^ (positive velocities towards the AP, negative velocities towards the VP). **B”**, Violin plots of the average velocity of bulk cytoplasmic flows, measured in areas parallel (green, *N* = 3 animals, *n* = 4 oocytes) and perpendicular to the oocyte AV axis (blue, *N* = 3 animals, *n* = 4 oocytes), and cortical actin flows along the oocyte AV axis (red, *N* = 4 animals, *n* = 4 oocytes). Solid lines indicate median. Dashed lines indicate quartiles. One-way ANOVA test with’Tukey’s multiple comparisons test. Unfertilized: *P* = 0.7841 for AV axis-LR axis, *P* = 0.6321 for AV axis-Actin and *P* = 0.6553 for LR axis-Actin; 1mpf: *P* = 0.7270; *P = 0.0227; **P = 0.0071; 3mpf: *P* = 0.9401; *P = 0.0412 for AV axis-Actin and *P = 0.0244 for LR axis-Actin; 6mpf: *P* = 0.0596; ***P = 0.0002; **P = 0.0068; 9mpf: *P* = 0.9871 for AV axis-LR axis, *P* = 0.4320 for AV axis-Actin and *P* = 0.3581 for LR axis-Actin; 13mpf: *P* = 0.4983 for AV axis-LR axis, *P* = 0.3306 for AV axis-Actin and *P* = 0.9382 for LR axis-Actin. ns, not significant.

Gradients of actin and/or myosin II can give rise to actomyosin flows^8,9,22^. We thus asked whether the unequal distribution of cortical actomyosin along the AV axis of the oocyte (Fig. 2a,b and Extended Data Fig. 3c,d, pink area) might lead to cortical actomyosin flows, which again drive CP formation. By using particle image velocimetry (PIV) and kymograph analysis to detect such flows in the oocyte, we found that unusually fast (8.2 ± 4.3 µm per min at 3 mpf) vegetal-directed cortical actomyosin flows were established upon oocyte fertilization. These flows were initially confined to the animal hemisphere of the fertilized oocyte and eventually extended along the entire AV axis (Fig. 2c and Extended Data Fig. 3f, pink area), leading to a strong accumulation of cortical actomyosin at the VP at the end of CP initiation (Fig. 2b and Extended Data Fig. 3d). During CP expansion, actomyosin flows ceased (Fig. 2c and Extended Data Fig. 3f, orange area), and actomyosin distribution along the AV axis of the oocyte nearly equalized again. Notably, cortical actin flows at fertilization and during CP formation were accompanied by the bulk cytoplasm close to the cortex flowing towards the VP (Extended Data Fig. 4a,b) and, as a result of these VP-directed flows, bulk cytoplasm in the centre of the oocyte flowing backwards from the VP towards the AP (Extended Data Fig. 4a,b′). However, these flows were considerably slower and shorter-ranged than those of the actin cortex, arguing against a decisive role of such flows in CP formation (Extended Data Fig. 4b′′).

To better understand how cortical actomyosin flows are established upon fertilization and how they might function in CP formation, we examined cortical actin dynamics at high temporal resolution (Fig. 2a,d and Supplementary Video 4). We found that before fertilization, an actin-rich patch, colocalizing with the position of the meiotic spindle at the AP, was surrounded by an area largely devoid of actin (Fig. 2e,e′, arrowhead). This indicates that the cortex is generally weaker at the AP compared to the rest of the oocyte surface and, therefore, that the AP is more susceptible to contractility-induced cortical instabilities^1,23^. Upon fertilization, a second small zone of local actin depletion appeared (Fig. 2a, asterisk), likely corresponding to the sperm entry point, given that it appeared only upon fertilization^24,25^ and preceded any recognizable deformation of the oocyte. Concomitant with the elongation of the oocyte along its AV axis upon fertilization, we found that the actin cortex opened at the actin-depleted region of the AP, and that the size of this opening rapidly increased thereafter (Fig. 2a, dashed line, and Fig. 2d, dashed arrows). This rapid expansion of the cortical opening at the AP led to the formation of two distinct cortical actin regions: a zone of low cortical actin density at the AP outlining the opening of the actin cortex, and a region of high actin density covering the remainder of the zygote surface (Fig. 2a,d, 1 min 20 s). With the expansion of the cortical opening proceeding, the high-actin-density region became further restricted to the vegetal half of the oocyte and progressively constricted towards the VP until the end of CP initiation (Fig. 2a, arrow). This was also when cortical flows had ceased, cortical actin had accumulated almost entirely at the VP, and the VP had transiently retracted and formed a small bulge (Fig. 2a,d, 3 min 12 s, arrowhead). Collectively, these results indicate that CP initiation is characterized by fertilization-induced cortical actomyosin contraction and flows towards the VP.

## Increased cortical tension leads to cortex opening and flow

**Fig. 3:**
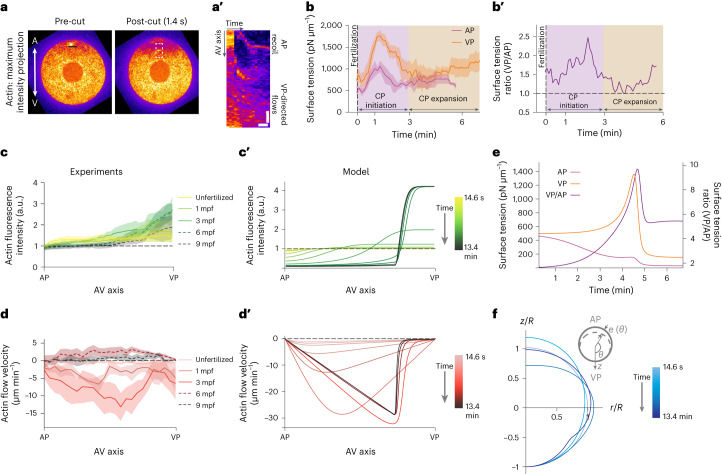
Increased cortical tension upon fertilization leads to cortex opening and flow. **a**, Confocal fluorescence images of maximum intensity projections of actin (Utrophin-Venus) intensity at the AP in an exemplary unfertilized oocyte before and after UV-laser ablation. Line demarcates the ablation site. Scale bar, 30 µm. **a′**, Kymograph along the dashed rectangle in **a** of cortical actin recoil and flow at the AP as a function of time after UV-laser ablation. Vertical scale bar, 10 µm. Horizontal scale bar, 3 s. **b**, Plot of measured oocyte surface tension at the AP (pink line) and VP (orange line) as a function of time after fertilization (0 min, dashed vertical line) (*N* = 3 animals, *n* = 3 oocytes for AP; *N* = 3 animals, *n* = 5 oocytes for VP). Error bars, s.e.m. **b′**, Plot of the ratio of the vegetal to animal surface tension over time after fertilization (0 min, dashed vertical line). Data from **b**. **c**,**c′**, Plot of average actin fluorescence intensity projected along the AV axis during CP initiation (solid lines, unfertilized, 1 mpf and 3 mpf) and CP expansion and absorption (dashed lines, 6 mpf and 9 mpf) measured experimentally (*N* = 4 animals, *n* = 4 oocytes) (**c**) and predicted in our model (**c′**), with friction $\xi =1.0\eta {R}^{2}$. In **c**, error bars, s.e.m. **d**,**d**′, Plot of average actin flow velocity projected along the AV axis during CP initiation (solid lines, unfertilized, 1 mpf and 3 mpf) and CP expansion and absorption (dashed lines, 6 mpf and 9 mpf) measured experimentally from kymographs as in Fig. 2c (*N* = 4 animals, *n* = 4 oocytes) (**d**) and predicted in our model with friction $\xi =1.0\eta {R}^{2}$ (**d′**). In **d**, error bars, s.e.m. **e**, Plot of the evolution of surface tension at the VP (orange line), AP (pink line) and their ratio (purple line) predicted in our model with friction $\xi =1.0\eta {R}^{2}$. **f**, Theoretical model of cell contour changes resulting from cortical accumulation and flow over time. For convenience, only one side is shown (the cell is symmetric around *z*). Source data

**Extended Data Fig. 5:**
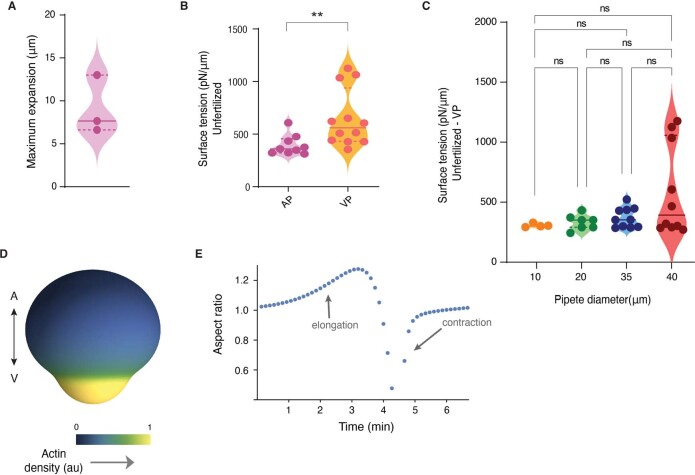
Surface tension of the unfertilized oocyte along the AV axis. **A**, Violin plot of the maximum expansion/opening of the actin cortex at the animal pole following UV-laser ablation. *N* = 3 animals, *n* = 3 oocytes. Solid line indicates the median, dashed lines indicate the quartiles. **B**, Violin plots of the surface tension at the AP (*N* = 6 animals, *n* = 9 oocytes) and VP (*N* = 5 animals, *n* = 12 oocytes) of unfertilized oocytes. Solid lines indicate the median, dashed lines indicate the quartiles. Two-sided Mann-Whitney test (**P = 0.0056). **C**, Violin plots of the surface tension at the VP of unfertilized oocytes determined with different pipette diameters: 10 µm (*N* = 3 animals, *n* = 4 oocytes), 20 µm (*N* = 4 animals, *n* = 7 oocytes), 35 µm (*N* = 4 animals, *n* = 10 oocytes), 40 µm (*N* = 4 animals, *n* = 10 oocytes). Solid line indicates the median, and the dotted line indicates the quartiles. Kruskal-Wallis test with Dunn’s multiple comparison test (ns, not significant, *P* > 0.9999 for 10 µm-20 µm, 10 µm-35 µm, 10 µm-40 µm, 20 µm-35 µm, 20 µm-40 µm and 35 µm-40 µm; *P* = 0.1403 for 35 µm-40 µm). **D**, Oocyte shape model prediction of a bulge near the VP associated with the steep gradient in cortical actin density. Color bar indicates actin density in arbitrary units. See text for choice of model parameters. **E**, Plot of the AR during CP initiation and expansion predicted by the model with friction $\xi =1.0\eta {R}^{2}$. See text for choice of model parameters. Source data

Given the coincidence of cortical opening at the AP and the initiation of fast vegetal-directed retraction and flow of the actomyosin cortex upon fertilization, we speculated that cortical opening might initiate cortical flows towards the VP. To test this hypothesis, we performed ultraviolet (UV)-laser ablations of the actin cortex at the AP of unfertilized oocytes with the aim of mimicking the tear of the cortex that occurs upon fertilization (Fig. 3a,a′, Extended Data Fig. 5a and Supplementary Video 5). We found not only that the AP expanded upon ablation (Extended Data Fig. 5a), similar to what was observed during fertilization-induced opening of the actin cortex at the AP (Figs. 1a and 2a, pink area), but also that cortical actin flowed towards the VP in the vicinity of the cut (Fig. 3a′). Notably, the restriction of these flows to the vicinity of the cut and their fast relaxation at the scale of seconds indicate that the cortex is stable before fertilization, when increased cortical contractility destabilizes the cortex, leading to faster cortical opening and more global actomyosin flows (Fig. 2a,c,d, pink area). To directly probe how the cortical stress pattern changes upon fertilization, we used micropipette aspiration to monitor changes in cortical tension during fertilization (for technical details and control experiments, see Methods and Extended Data Fig. 5b,c). We found that cortical tension was higher at the VP than the AP in unfertilized oocytes (Extended Data Fig. 5b), and that this difference strongly increased upon fertilization until 1 mpf (Fig. 3b, pink area), coinciding with the opening of the cortex at the AP (Fig. 2a,d, 48 s). Moreover, cortical tension heterogeneity reached its maximum just before the end of CP initiation (Fig. 3b′), when the actomyosin cortex had accumulated almost entirely at the VP (Figs. 2a,d, 3 min 48 s). Collectively, these findings support the notion that an increase in contractility along the oocyte AV axis upon fertilization leads to cortical instabilities and opening of the cortex at its weakest point at the AP, resulting in fast retraction of the cortex directed towards the VP.

To understand the extent to which the observed cortical actomyosin flows can explain the oocyte shape changes occurring during CP formation, we developed a minimal theoretical model of this process based on active gel theory^26–28^. Although in general the cortex is depicted as viscoelastic, we assumed that it behaves as a viscous fluid on experimental timescales (~minutes), consistent with PIV measurements showing large in-plane displacements and following earlier models of cortical dynamics^9^. In this description, the cortical dynamics are given by the (two-dimensional) actin density $\rho (\theta ,t)$ and the velocity field $\upsilon \left(\theta ,t\right)$ describing flow along a meridional line connecting the VP $(\theta =0)$ and the AP $(\theta =\uppi )$. Here, $\theta$ is the polar angle measured relative to the VP such that *z* = *θ* represents the AV axis. In a first step, assuming axi-symmetry around the AV axis, we determined the cortical tensions by solving the equations of in-plane force balance and cortical mass conservation for $\upsilon$ and $\rho$ on an undeformed reference sphere of radius $R$ representing the unfertilized oocyte. In a next step, we used these tensions to determine the cell shape by solving the equation of out-of-plane force balance or, equivalently, force balance along the symmetry (AV) axis.

The tensions along $\left(\theta \right)$ and perpendicular $\left(\phi \right)$ to a meridional line depend on density and flow via^29^

1 $$\begin{array}{c}{T}_{\theta }={T}_{0}\left(\,\rho \right)+2\eta {R}^{-1}{\partial }_{\theta }\upsilon +\gamma {\nabla }^{2}\rho \\ {T}_{\phi }={T}_{0}\left(\,\rho \right)+2\eta {R}^{-1}\cot \theta \upsilon +\gamma {\nabla }^{2}\rho \end{array}$$

In these equations, the first term ${T}_{0}\left(\rho \right)$ is the isotropic tension, including the effect of contractility. The second term arises from viscosity $\eta$, and the third term penalizes abrupt spatial changes in density with coefficient $\gamma$. The in-plane force balance and mass conservation equations are given by

2 $$\begin{array}{c}{\partial }_{\theta }{T}_{\theta }+{\mathrm{cot}}\theta ({T}_{\theta }-{T}_{\phi })=\xi R\upsilon \\ {\partial }_{t}\rho +\nabla (\,\rho \upsilon )=-{k}_{d}(\,\rho -{\rho }_{0})\end{array}$$

where ${k}_{d}$ is the cortical turnover rate and ${\rho }_{0}$ the cortical density before fertilization. In the above, $\xi$ is an effective friction coefficient that accounts for the interactions between the cortex and adjacent cytoplasm. We note that, in general, $\xi$ could be $\theta$-dependent, but because including an angle dependence did not qualitatively change the results given below, we assumed that $\xi$ is constant.

The dependence of ${T}_{0}$ on density governs how an initial weakening of the cortex near the AP during fertilization will destabilize the cortex, leading to flows, accumulation of material near the VP, and CP initiation. Based on previous studies showing that myosin activity can drive contractile instabilities in the cortex^1,30,31^, we write

3 $${T}_{0}\left(\,\rho \right)={T}_{\text{m}}-a\rho +\zeta {\rho }^{2}-b{\rho }^{3}$$

Here, ${T}_{\text{m}}$ is a constant representing the density-independent part (membrane part) of the tension. The constants $a$, $b$ and $\zeta$ are positive constants, with $\zeta$ representing myosin-induced contractility. This minimal choice of functional dependence on $\rho$ leads to a bistability of actin enrichment in the cortex. The term $\zeta {\rho }^{2}$ will tend to destabilize a uniform cortex, whereas the terms with coefficients $a$ and $b$ will stabilize states with low and high actin density, respectively. Consequently, for contractilities $\zeta > \sqrt{3{ab}}$, a uniform cortex with density ${\rho }_{-} < \rho < {\rho }_{+}$, where ${\rho }_{\pm }=\frac{\zeta }{3b}\left(1\pm \sqrt{1-\frac{3{ab}}{{\zeta }^{2}}}\right)$, becomes unstable.

To connect our model with experiments, we represented fertilization as an increase of contractility (as indicated by the observed upregulation of contractility) and a local drop in actin density near the AP $(\theta =\uppi )$, mimicking the observed cortex opening. We assumed that the cortex is stable before fertilization, with density $\rho > {\rho }_{+}$, and that fertilization at time $t=0$ brings the cortex into the unstable regime, with a density $\rho ={\rho }_{0}$ everywhere except in a small area near $\theta =\uppi$. To find the cortical dynamics and oocyte shape change, we chose a representative set of parameters based on quantities measured experimentally and taken from literature (Methods and Supplementary Information).

Solving equations (1)–(3) with appropriate boundary conditions (see Supplementary Information for details), we found that the model qualitatively captured the observed cortical behaviour during CP initiation (Fig. 3c,c′,d,d′, solid lines; note that the cortical behaviour during CP expansion and absorption—dashed lines—are not covered by this model). As shown in Fig. 3c′ (density) and Fig. 3d′ (velocity), instability and a small drop in cortical density near the AP triggered a flow and resultant accumulation of cortex material towards the VP. Over time, the flow amplitude increased and its maximum shifted towards the VP, consistent with the experimentally measured cortical flow profile during CP initiation (Fig. 3d, solid lines). Notably, the steady-state actin flow profile that we obtained with the model shows a more asymmetric profile than the experimental flow profile in Fig. 3d,d′, probably because of our choice of a simple rheological model for the cortex.

With the model density and velocity profiles known, the corresponding cortical tensions can be calculated (equations (1) and (3)) and compared with our measurements: following fertilization, the tension at the VP was always greater than at the AP, consistent with cortical flow towards the VP (Fig. 3e). Furthermore, both tensions peaked and then decreased before tending to values before fertilization (Fig. 3e). These model predictions agree with the salient features of the total tension, $\frac{1}{2}\left({T}_{\theta }+{T}_{\phi }\right)$ at the VP and AP found experimentally (Fig. 3b,b′).

We next sought to understand how changes in cortical tension affect oocyte shape during CP initiation. To this end, we applied a perturbative approach: the tensions calculated above in a spherical reference state were used as input in Laplace’s law to find the radial cortical displacement $e\left(\theta ,t\right)$, which is assumed to be small compared with *R*^29,32,33^. We note that numerical methods that allow for simultaneous and coupled solutions of arbitrarily large in-plane tensions and out-of-plane displacement exist^27,34,35^. However, we have opted here for the simpler analytical approach, as it contains fewer unknown parameters while still yielding physical insight into the experimentally determined oocyte shape. Assuming axi-symmetry around the AV axis, to leading order in *e*/*R*, the change in total curvature from the reference curvature $2/R$ is related to the change in cortical tension $\delta {T}_{\theta }+\delta {T}_{\phi }\equiv {T}_{\theta }+{T}_{\phi }-2{T}_{0}\left(\,{\rho }_{0}\right)$ as

4 $$e^{{\prime}{\prime} }(\theta )+\cot \theta {e}^{{\prime} }\left(\theta \right)+2e\left(\theta \right)=\frac{R}{{T}_{0}\left(\,{\rho }_{0}\right)}(\delta {T}_{\theta }+\delta {T}_{\phi }-R\delta p)$$

In this equation, $\delta p$ represents a normal cytoplasmic stress needed to maintain the oocyte volume and ensure that the overall force on the oocyte along $z$ is zero (assuming it is isolated; see Supplementary Information for details). Importantly, in the presence of friction on the cortex caused by interaction with the adjacent cytoplasm, $\delta p$ is non-uniform, being greater near the VP than the AP. Thus, equation (4) is a non-trivial expression of Laplace’s law and consistent with observations that cortical tension over the radius of curvature is greater at the VP than at the AP during CP initiation (Fig. 3b,b′). From the numerical solution to equation (4) for $e\left(\theta \right)$, the model cell shapes can be reconstructed (Fig. 3f). The model qualitatively reproduced the experimental sequence of oocyte shape changes during CP initiation (Fig. 1a, pink area): first an elongation along the AV axis, followed by a contraction and a pinching of the surface in the vegetal half (Fig. 2a,d, arrows). The pinching occurred where the gradient in density is steepest (Fig. 2d, arrows and Extended Data Fig. 5d) and can be attributed to the effective line tension $\gamma$. Calculation of the AR as a result of this elongation–contraction behaviour, with ${{\mathrm{AR}}}=\frac{(R+e\left(\pi \right))}{\left(R+e\left(\frac{\pi }{2}\right)\right)}$, qualitatively agreed with the experimental AR measurements during CP initiation (Extended Data Fig. 5e), supporting the notion that the rapid elongation and then sudden contraction and pinching of the surface in the vegetal half are caused by changes in cortical tension at the VP. Collectively, the model, in qualitative agreement with experimental observations, provides a mechanistic explanation of how cortical dynamics, and in particular its coupling to cell bulk by means of frictional forces, can lead to oocyte shape changes during CP initiation.

## The myoplasm mechanically interacts with the actin cortex

**Fig. 4:**
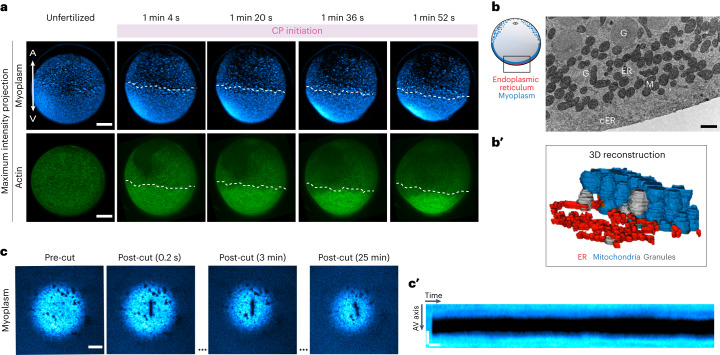
A mitochondria-rich subcortical layer overlaps with the high-actin-density domain during CP initiation. **a**, Confocal fluorescence images of maximum intensity projections of the myoplasm (MitoTracker Deep Red FM) (top row) and cortical actin (Utrophin-Venus) (bottom row) in an exemplary oocyte during consecutive stages of CP initiation. Dashed line demarcates the edge of the compacted myoplasm layer in the vegetal hemisphere of the oocyte. Scale bar, 30 µm. **b**, EM section of an exemplary region in the vegetal hemisphere of an unfertilized oocyte (outlined in the schematics on the left). Note the ER sheets in the myoplasm. Scale bar, 1 µm. **b′**, Three-dimensional reconstruction of 50 sections of 70 µm taken in the same region shown in **b**. Mitochondria, blue; cER, red; granules, grey. For clarity, only cER from the first sections is shown. **c**, Confocal fluorescence images of the myoplasm (MitoTracker Deep Red FM) at the VP in an exemplary unfertilized oocyte before and after UV-laser cutting. Scale bar, 15 µm. **c′**, Kymograph of myoplasm recoil following UV-laser cutting as a function of time after the cut. Horizontal scale bar, 5 sec. Vertical scale bar, 2 µm. 3D, three-dimensional; M, mitochondria; G, granules. Source data

Although our findings so far explain how actin cortex contraction and flow upon fertilization can lead to the oocyte shape changes observed during CP initiation, questions remain as to the processes leading to subsequent CP expansion and eventual absorption. To address this question, we asked whether cytoplasmic structures other than the actin cortex might be involved in these processes. Ascidian oocytes contain a mitochondria-rich subcortical layer called ‘myoplasm’ that has been proposed to accumulate at the CP and play a role in axis establishment during oocyte maturation and early embryonic development^17^. Consistent with previous findings^17^, maximum intensity projections of the myoplasm during early stages of CP initiation showed that in the animal half of the oocyte, the myoplasm was organized into small and highly dispersed clusters, visualized by their high mitochondria content; in the vegetal half, it appeared as a compacted thick layer deposited along the actin cortex (Fig. 4a, top row). Interestingly, this compacted region of myoplasm (Fig. 4a, white dotted line) colocalized with the high-actin-density domain in the vegetal half of the oocyte during the first stages of CP initiation (Fig. 4a, bottom row), pointing to the possibility that subcortical myoplasm might functionally interact with the adjacent cortex.

To determine whether and how subcortical myoplasm might interact with the overlying actin cortex, we first used transmitted electron microscopy (EM) to visualize the spatial association of cortex and myoplasm. As reported previously^17^, we found that the mitochondria-rich myoplasm is deposited over the cER in close proximity to the cortex (Fig. 4b). Interestingly, three-dimensional reconstruction analysis of 50 serial thin-section EM images of the VP of unfertilized oocytes revealed that the mitochondria in the myoplasm do not form an interconnected mitochondrial network as in other systems^36,37^, but that the myoplasm is formed by densely packed individual mitochondria (Fig. 4b′), similar to what has been reported in the Balbiani body of *Xenopus* and zebrafish oocytes^38,39^.

**Extended Data Fig. 6:**
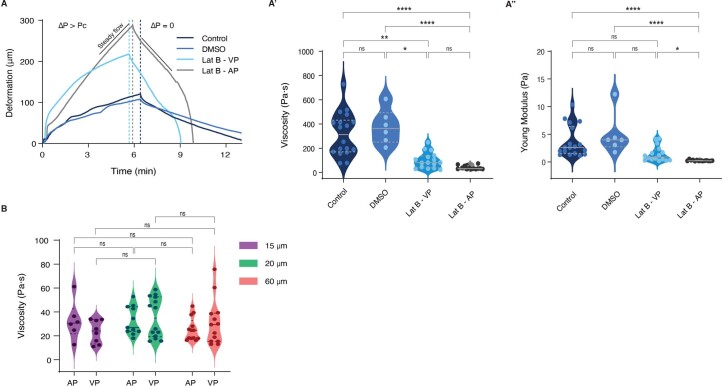
Viscosity and elasticity of the myoplasm. **A**, Exemplary deformation curves of the aspirated oocyte in the aspiration experiments for untreated (control) and DMSO-treated (0.1%) oocytes at the VP, and LatB-treated (1 µg ml^−1^) oocytes at the AP and VP. Solid line indicates the slope of the linear steady flow of the oocyte into the pipette. Dashed vertical lines indicate the time point of pressure change. ∆P, aspiration pressure (150 Pa for control and DMSO, 100 Pa for LatB at the VP and 50 Pa for LatB at the AP). Pc, critical pressure. **A’** and **A”**, Violin plots of viscosity (**A’**) and Young Modulus (**A”**) calculated from the creep experiments (aspiration curves only) shown in (**A**). Solid lines indicate median. Dashed lines indicate quartiles. Control, *N* = 8 animals, *n* = 18 oocytes; DMSO, *N* = 3 animals, *n* = 6 oocytes; LatB - VP, *N* = 8 animals, *n* = 16 oocytes; LatB - AP, *N* = 3 animals, *n* = 14 oocytes. Kruskal-Wallis test with Dunn’s multiple comparison test. **A**’, *P* > 0.9999 for Control-DMSO and *P* = 0.2747 for LatB VP-LatB AP; *P = 0.0157; **P = 0.0056; ****P < 0.0001. **A**”, *P* > 0.9999 for Control-DMSO, *P* = 0.1016 for Control-LatB VP and *P* = 0.0835 for DMSO-LatB VP; *P = 0.0301; ****P < 0.0001. **B**, Violin plots of viscosity values (obtained from aspiration curves only) of the AP and VP of unfertilized oocytes treated with 1 µg ml^-1^ LatB measured with pipettes of different sizes (purple, 15 µm, *N* = 4 animals, *n* = 6 oocytes (AP) and 8 oocytes (VP); green, 20 µm, *N* = 4 animals, *n* = 11 oocytes (AP) and 14 oocytes (VP) and red, 60 µm, *N* = 4 animals, *n* = 13 animals (AP) and 12 animals (VP)). Solid lines indicate median. Dashed lines indicate quartiles. Kruskal-Wallis test with Dunn’s multiple comparison test. *P* > 0.9999 for all comparisons. ns, not significant. Source data

Notably, the Balbiani body in zebrafish and *Xenopus* has been described as a membrane-less amyloid-like condensate with solid-like properties^40^. To directly test whether the myoplasm of ascidian oocytes also behaves as a solid-like layer, we performed UV-laser ablations of the myoplasm in unfertilized oocytes (Fig. 4c,c′ and Supplementary Video 6). Consistent with it behaving like a relaxed solid before fertilization, the myoplasm did not show any recognizable recoil upon laser cutting (Fig. 4c, post-cut, 0.2 s), and the cut did not close following the ablation (Fig. 4c, post-cut, 25 min and Fig. 4c′). We also performed creep experiments using micropipette aspiration at the AP and VP of unfertilized oocytes and oocytes treated with 1 µg ml^−1^ LatB to weaken the actin cortex (Extended Data Fig. 6a,a′′; for technical details and control experiments, see Methods and Extended Data Fig. 6b). This showed that both viscosity and elasticity at the VP were substantially reduced in LatB-treated versus control oocytes (Extended Data Fig. 6a′,a′′), indicating that the myoplasm at the VP displays a smaller viscosity and elasticity than the actin cortex. Moreover, elasticity was significantly higher at the VP than the AP, indicating that the myoplasm at the VP increases elasticity in this region (Extended Data Fig. 6a′′). Together, this supports the notion that the myoplasm behaves as a viscoelastic solid that might mechanically interact with the overlying actin cortex at the vegetal hemisphere of the oocyte.

## The myoplasm buckles during CP initiation

**Fig. 5:**
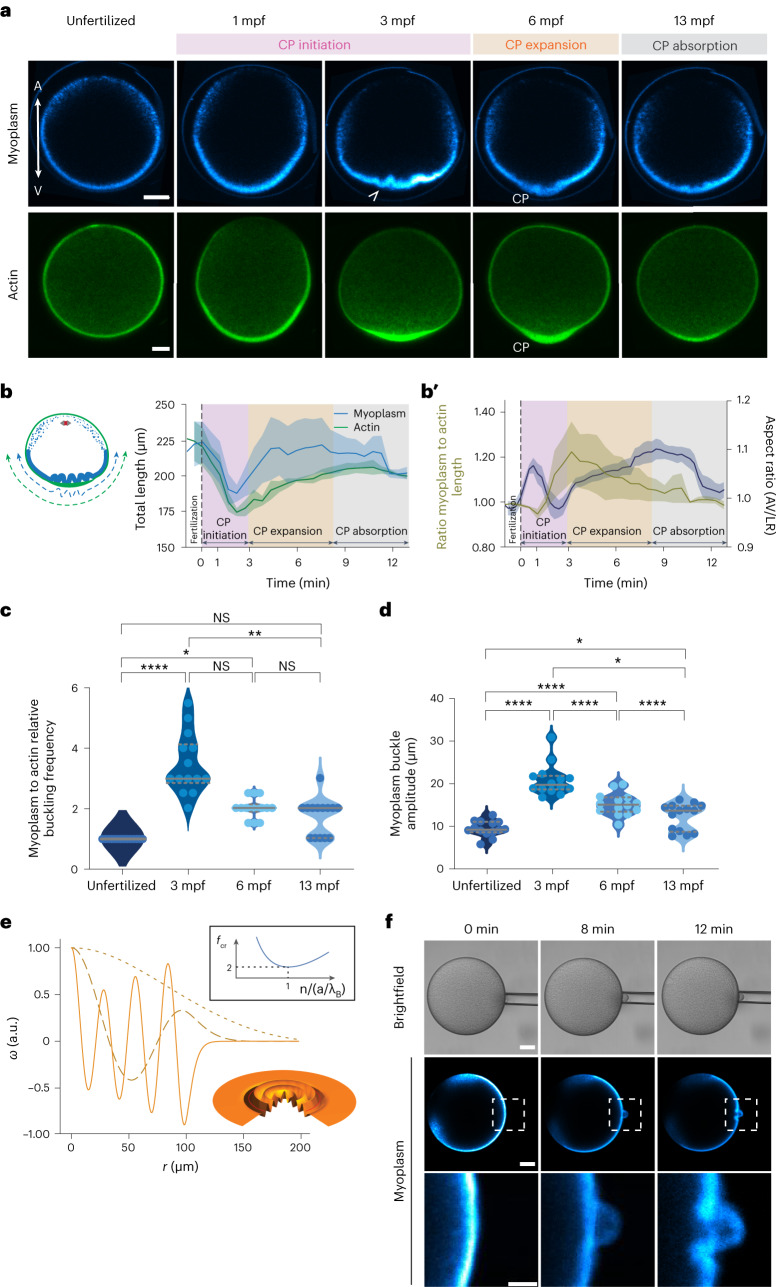
Changes in myoplasm buckling frequency during CP initiation and expansion. **a**, Confocal images of sum intensity projections over 10 central z-slides of the myoplasm (MitoTracker Deep Red FM, top row) and cortical actin (Utrophin-Venus, bottom row) during CP morphogenesis. Arrowhead points at myoplasm buckles. Scale bar, 30 µm. **b**, Total length of the compacted layer of myoplasm (blue) and the underlying actin cortex (green) as a function of time after fertilization (0 min, dashed vertical line). (*N* = 4 animals, *n* = 5 oocytes.) Error bars, s.e.m. **b′**, Ratio of myoplasm to actin length (yellow) and the AR of the oocyte (blue; from Fig. 1b) as a function of time after fertilization (0 min, dashed vertical line). Error bars, s.e.m. **c**, Violin plots of the buckling frequency of the myoplasm relative to the actin cortex before fertilization (unfertilized) and at 3, 6 and 13 mpf. (*N* = 9 animals, *n* = 14 oocytes.) Solid lines, median. Dashed lines, quartiles. One-way Friedman test and Dunn’s multiple comparisons test (*P* = 0.2027 for unfertilized, 13 mpf; *P* = 0.0625 for 3–6 mpf and *P* > 0.9999 for 6–13 mpf; *P = 0.0127 for unfertilized 6 mpf; **P = 0.0027; ****P < 0.0001). **d**, Violin plots of the buckling amplitude of the myoplasm before fertilization (unfertilized) and at 3, 6 and 13 mpf. (*N* = 9 animals, *n* = 14 oocytes.) Solid lines, median. Dashed lines, quartiles. One-way analysis of variance and Holm–Sidak multiple comparisons test (*P = 0.0184, ****P < 0.0001). **e**, Plot of buckling of an elastic disk embedded in media of different elasticity (decreasing stiffness from solid to dashed to dotted curves) by friction forces concentrated at a distance *r* = *a*. Wavelength of vertical deflection, $w$, as a function of *r* (measured from VP; *r* = *0*) near the buckling. Inset: dependence of threshold force on rescaled mode number (buckling frequency). **f**, Brightfield (top row), confocal (middle row) and high-magnification (bottom row) images of the myoplasm in unfertilized oocytes during micropipette aspiration. Scale bar, 30 µm. NS, not significant. Source data

**Extended Data Fig. 7:**
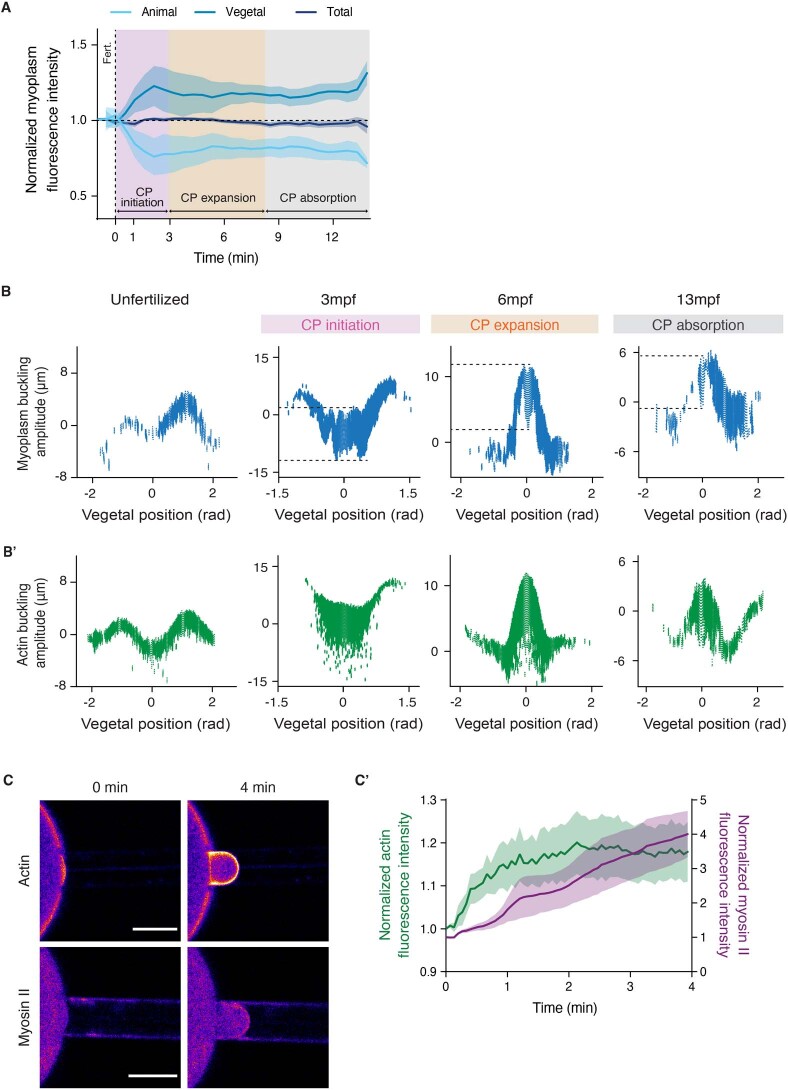
Myoplasm dynamics during CP morphogenesis. **A**, Plot of myoplasm (MitoTracker Deep Red FM) fluorescence intensity in oocytes as a function of time after fertilization (0 min, dashed vertical line) for the entire oocyte (total intensity) and within the animal or vegetal hemispheres of the oocyte (*N* = 4 animals, *n* = 6 oocytes). Error bars, s.e.m. **B** and **B’**, Buckling amplitude for myoplasm (**B**) and cortical actin (**B’**) as a function of their position relative to the CP (fixed at 0 radians) before fertilization (unfertilized) and at 3, 6 and 13 min post fertilization (mpf). The horizontal dashed lines outline the buckling amplitude of the myoplasm. **C**, Confocal images of actin (Utrophin-Venus, top row) and myosin II (iMyo-YFP, bottom row) of unfertilized oocytes during micropipette aspirations as in Fig. 5e. Scale bar, 30 µm. **C’**, Plot of normalized actin (green, left y-axis) and myosin II (magenta, right y-axis) fluorescence intensities as a function of time during the aspiration experiment shown in (**C**). *N* = 3 animals, *n* = 4 oocytes for each condition. Error bars, s.e.m. Source data

The tight spatial association of the flowing actin cortex with the more stationary myoplasm at the vegetal hemisphere of the oocyte points at the possibility that friction forces may arise at their interface. Such friction forces may not only resist cortical actin flows towards the VP but also trigger some deformation of the myoplasm. To address this possibility, we first estimated the degree of friction at this interface based on our theoretical description of the experimentally observed actin flow profiles during CP initiation. Interestingly, obtaining actin flow profiles matching the experimentally observed required setting the friction length^9^, $l=\sqrt{\frac{2\eta }{\xi }}$, no greater than of the order of $R$, indicating that friction forces are typically smaller than viscous forces in the cortex and, thus, only mildly affect cortical flow patterns during CP initiation and formation. To determine whether such friction forces might nevertheless affect the myoplasm, we analysed how the myoplasm reorganizes during CP formation (Fig. 5a and Supplementary Video 7). Similar to actin and myosin II, myoplasm fluorescence intensity decreased at the animal and increased at the vegetal hemisphere of the oocyte shortly after fertilization (Extended Data Fig. 7a, pink area), indicative of some vegetal-directed relocalization/flows of loosely associated myoplasm at the animal hemisphere towards the vegetal hemisphere that spatiotemporally correlate with the vegetal-directed flows of actin and myosin II. We also observed that upon arrest of cortical actin flows and the end of CP initiation (3 mpf), the compacted myoplasm layer at the vegetal hemisphere folded in a pattern resembling buckling of tissues under confinement/compression^41,42^ (Fig. 5a, 3 mpf, arrowhead). Buckles formed all along the compacted myoplasm layer and were more pronounced at the point of maximum actin accumulation where the CP formed. Measuring the total length of the compacted myoplasm and the underlying actin cortex and calculating their ratio confirmed that the myoplasm, but not the actin cortex, buckles close to the oocyte VP (Fig. 5b,b′).

Buckling is an example of an elastic instability in materials subjected to a compressive force that becomes evident by the appearance of deformations orthogonal to the main compression axis^43^. Considering that the number and amplitude of buckles reached their maximum at the end of CP initiation, when the actin cortex had almost entirely accumulated at the VP (Fig. 5c,d and Extended Data Fig. 7b,b′), those buckles are likely caused by the vegetal-directed flow of the actin cortex compressing the myoplasm through friction forces generated at the myoplasm–cortex interface. Using a minimal model, we tested whether the myoplasm buckling behaviour is due to a finite-wavelength buckling instability as a result of compressive friction forces.

To this end, the myoplasm was treated as a shallow elastic shell with a bending modulus $B$ and radius $R$ in contact with the cortex. The shallowness assumption is justified on the basis of the observation that buckling occurs near the VP and on a length scale small compared with the cell radius (Fig. 5c and Extended Data Fig. 7b). The external, frictional force density on the myoplasm was assumed proportional to the local actin cortical velocity, which is a non-monotonic function with a peak centred between the cell equator and the VP (Fig. 3d,d′). Furthermore, we assumed that the myoplasm is linked to the adjacent cytoplasm, likely through the cER^44^. To keep the model analytically tractable and to obtain physical insight, we also assumed an effective delta-function tangent force density at a position $r=a$, with $r=0$ coinciding with the VP and $r=R$ the myoplasm edge. We next simplified the initial state of the myoplasm to that of a disk (the effect of its initial curvature is discussed in the Supplementary Information). Denoting $w(r,t)$ the deflection of myoplasm, force and torque balances lead to the following equations, assuming $w$ is small (Supplementary Information):

5 $$\begin{array}{c}-\varGamma {\partial }_{t}w=B{\nabla }^{4}w+f\,{\nabla }^{2}w+{Kw},r\le a\\ -\varGamma {\partial }_{t}w=B{\nabla }^{4}w+\left(\,f-\Delta f\,\right)\left[{\nabla }^{2}w+\frac{{R}^{2}}{{r}^{2}}\left({\partial }_{r}^{2}w-\frac{{\partial }_{r}w}{r}\right)\right]+{Kw},r > a\end{array}$$

In the above, $\varGamma$ is an out-of-plane friction coefficient, $B$ is the myoplasm bending stiffness and $K$ is the elastic resistance of the surrounding cytoplasm. The quantities $f=a\xi V\frac{1+\nu }{2}\left(1+\frac{1-\nu }{1+\nu }\frac{{a}^{2}}{{R}^{2}}\right)$ and $\Delta f=a\xi V\frac{1+\nu }{2}$, with $\nu$ the myoplasm’s Poisson ratio, reflect the tangential compressive force due to actin flow. A semi-analytical determination of the buckling threshold force, ${f}_{{{\mathrm{cr}}}}$, was obtained by solving the linear problem in equation (5) with ${\partial }_{t}w=0$, on enforcing continuity of $w$ and its first three space derivatives at $r=a$ and using the method of matched asymptotics (see Supplementary Information for details). Interestingly, we found that the deflection $w(r)$ near the threshold consists of short-wavelength ripples in the region $r\le a$ and an amplitude decaying rapidly for $r > a$ (Fig. 5e). This pattern qualitatively resembles the myoplasm shape near the onset of buckling, at 3 mpf (Fig. 5a and Extended Data Fig. 7b), indicating that our model constitutes a plausible framework for explaining myoplasm buckling.

With this model in hand, we next asked whether the experimentally determined cortical flow would be sufficient to drive myoplasm buckling. Using values of parameters estimated from experimental observations, we predicted the buckling length scale caused by cortical flows to be ${\lambda }_{B}\approx 1 \upmu{\text{m}}$, taking estimated values for $B\approx {E}_{{\text{myo}}}{h}_{{\text{myo}}}^{3}$ and $K\approx {E}_{{\text{cyto}}}/R$, with ${E}_{{\text{myo}}}$ the myoplasm Young’s modulus, ${h}_{{\text{myo}}}$ its thickness and ${E}_{{\text{cyto}}}$ the cytoplasm Young’s modulus (see Supplementary Information for details). This closely matches the experimental value of the wavelength (Fig. 5d), indicating that cortical flow is sufficient to explain the buckling length scale of the myoplasm. We also predicted the critical value of the friction coefficient ${\xi }_{{\text{cr}}}$ to be in the order of $\sqrt{{BK}}/{aV}\approx 0.5$ Pa.s μm^−1^, assuming $B\approx 10$ Pa.μm^3^, $K\approx 1$ Pa μm^−1^ and $a\approx 50$ μm, a value that is on the order of the friction coefficient estimated from the experimentally obtained cortical flow damping: $\eta h/{R}^{2}\approx 1$ Pa.s μm^−1^. Collectively, this close match between theoretical prediction and experimental observation supports the notion that myoplasm buckling is caused by cortical flow friction.

To also challenge this notion experimentally, we ectopically induced cortical actomyosin flows locally in the vegetal hemisphere of unfertilized oocytes by micropipette aspiration and monitored whether this can induce local buckling of the adjacent myoplasm (Fig. 5f and Supplementary Video 8). When using a pipette with a small opening (20 μm in diameter) and applying a constant pressure below the critical pressure required to overcome the cortical surface tension, cortical actin and myosin II flowed into the aspiration pipette, whereas the myoplasm remained largely outside of it (Fig. 5f and Extended Data Fig. 7c,c′). Interestingly, the myoplasm directly adjacent to the aspiration site buckled (Fig. 5f, middle and bottom rows), indicative of cortical actomyosin flows into the aspiration pipette compressing the adjacent myoplasm through friction forces at their interface. This indicates that actomyosin flows at the VP are sufficient to induce myoplasm buckling.

Questions remain as to the potential function of myoplasm buckling in CP expansion. We observed that once the CP began expanding and protruding, VP-directed cortical flows ceased (Fig. 2c and Extended Data Fig. 3f) and cortical actin and myosin II accumulations at the VP diminished (Fig. 2b and Extended Data Fig. 3d), indicating that the cortical actomyosin network relaxes at the VP. Strikingly, this reduction in actomyosin at the VP was accompanied by the manifold buckles of the myoplasm resolving into one larger buckle outlining the expanded CP (Fig. 5a). This points to the intriguing possibility that the arrest of cortical flows and thus friction forces on the myoplasm together with the relaxation of the actin cortex at the onset of CP expansion at the VP cause the coalescence of buckles into a well-developed CP shape.

**Fig. 6:**
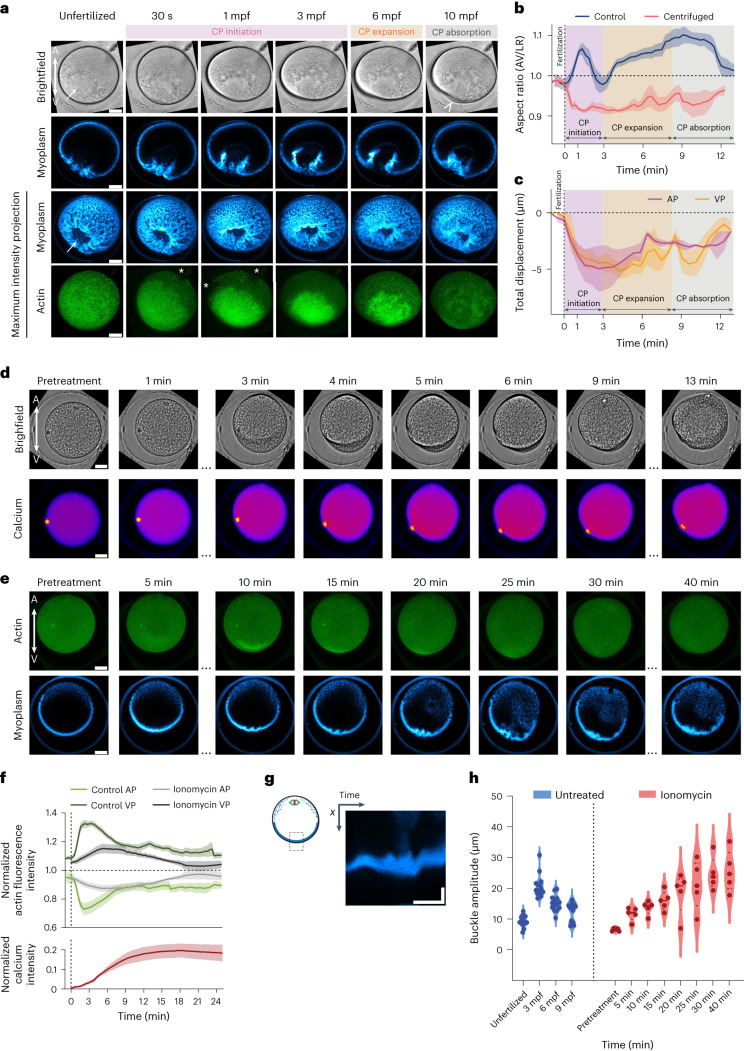
Interfering with the myoplasm impairs CP formation. **a**, Brightfield and confocal sum intensity projections over 10 central z-slides (15 µm) of the myoplasm (blue) and confocal maximum intensity projections of myoplasm and cortical actin (green) in an exemplary centrifuged oocyte during the first 10 min following fertilization. Arrows, displaced myoplasm; arrowhead, ‘pseudo-CP’; asterisks, actin cortex opening. Scale bar, 30 µm. **b**, Plot of AR of control (blue line, from Fig. 1b) and centrifuged oocytes (pink line) as a function of time after fertilization (0 min, dashed vertical line). *N* = 3 animals, *n* = 3 oocytes. Error bars, s.e.m. **c**, Plot of distance from the AP and VP to the CM in centrifuged oocytes as a function of time after fertilization (0 min, dashed vertical line). *N* = 3 animals, *n* = 3 oocytes. Error bars, s.e.m. **d**, Brightfield (top row) and confocal fluorescence images of sum intensity projections of intracellular Ca^2+^ (Calcium Green, bottom row) in a representative unfertilized oocyte at consecutive timepoints following Ionomycin exposure. Scale bar, 30 µm. **e**, Confocal maximum intensity projections of actin (Utrophin-mScalet, top) and a central confocal section of the myoplasm (MitoTracker Deep Red FM, bottom) in a representative unfertilized oocyte following Ionomycin exposure. Scale bar, 30 µm. **f**, Plot of total actin fluorescence intensity in the animal (AP) and vegetal (VP) hemispheres of control fertilized oocytes (green, top plot, from Fig. 2b), Ionomycin-treated unfertilized oocytes (grey, top plot, *N* = 4 animals, *n* = 5 oocytes) and Ca^2+^ fluorescence intensity (red, bottom plot, *N* = 4 animals, *n* = 5 oocytes). Error bars, s.e.m. **g**, Kymograph of the myoplasm at the vegetal-most part of the oocyte during Ionomycin treatment. x, width/myoplasm buckle amplitude. Horizontal scale bar, 15 min. Vertical scale bar, 15 µm. **h**, Violin plots of the buckling amplitude of the myoplasm during CP morphogenesis in control (blue, *N* = 9 animals, *n* = 14 oocytes) and Ionomycin-treated (red, *N* = 3 animals, *n* = 5 oocytes) oocytes. Source data

To test the plausibility of this assumption, we used our model for conceptualizing the evolution of the CP shape during the CP expansion. This showed that as the mean position at which friction acts, $a$, is much larger than ${\lambda }_{B}$, a certain number $n\approx a/{\lambda }_{B}$ of buckles are expected to form initially. This implies that the minimum of the critical buckling force ${f}_{{\text{cr}}}$ with respect to *n* is very shallow, and a large number of buckling modes can be excited (Fig. 5e, inset). However, at the transition of CP initiation to expansion, both *V* (flow speed) and *a* decrease, and therefore the average *n* decreases as well. Furthermore, once the flow stops (*V* = 0), the modes with the largest *n* will relax the fastest, leaving behind the longest-wavelength deformations. This behaviour matches our experimental observation of buckling resolution during CP expansion (Fig. 5a,c,d, 6 and 13 mpf), supporting the notion that upon arrest of friction forces, the relaxation dynamics of the myoplasm will lead to the myoplasm forming only a single buckle at large time scales (Fig. 5e, inset; see Supplementary Information for details).

**Extended Data Fig. 8:**
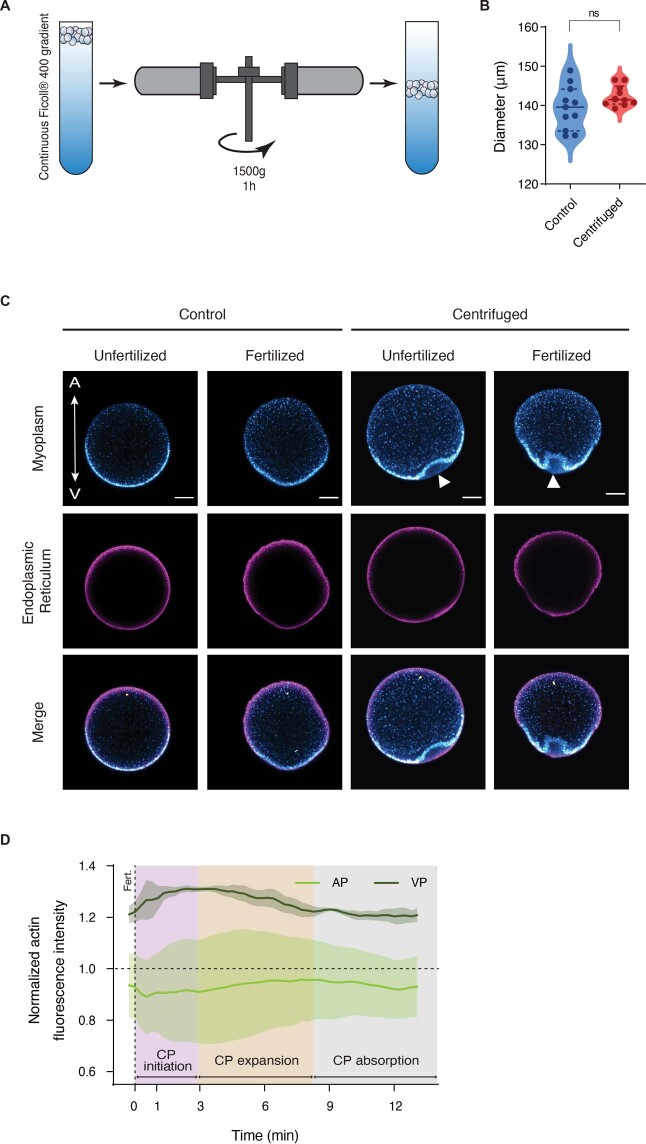
Oocyte centrifugation alters the position of the compacted myoplasm. **A**, Schematic representation of the centrifugation experiment. Taken from https://doi.org/10.15479/at:ista:9623. **B**, Violin plots of the diameter of control (blue bar. *N* = 5 animals, *n* = 11 oocytes) and centrifuged unfertilized oocytes (pink bar. *N* = 5 animals, *n* = 10 oocytes). Solid lines indicate median. Dashed lines indicate quartiles. Mann-Whitney test (n.s. is not significant, *P* = 0.1366). **C**, Confocal cross-sections of control and centrifuged oocytes immunostained for myoplasm (mouse anti-neurofilament antibody - see methods; blue), endoplasmic reticulum (rabbit anti-phospho-S6 antibody - see methods; magenta), and DNA (Hoechst; orange, merge). Arrowheads point at the displacement of the myoplasm from the vegetal-most cortex. Scale bar, 30 µm. **D**, Plot of total actin intensity in the animal (AP) and vegetal (VP) hemispheres of centrifuged oocytes as a function of time after fertilization (0 min, dashed vertical line). *N* = 3 animals, *n* = 3 oocytes. Error bars, s.e.m. Source data

To further experimentally challenge this notion, we first sought to interfere with myoplasm localization at the VP and monitor how this affects CP formation. To this end, we centrifuged unfertilized oocytes with the aim of displacing the myoplasm from the cortex at the vegetal oocyte hemisphere (Extended Data Fig. 8a) without affecting the shape and/or size of the centrifuged oocytes (Fig. 6a and Extended Data Fig. 8b). In a fraction of the centrifuged oocytes, the myoplasm was clearly displaced away from the vegetal cortex towards the oocyte interior (Fig. 6a, arrows, and Extended Data Fig. 8c). The actomyosin cortex and associated ER, in contrast, remained at the vegetal surface of those oocytes (Extended Data Fig. 8c), indicating that centrifugation specifically affected myoplasm subcortical localization. Upon fertilization, oocytes with displaced myoplasm at their VP failed to form a normcal CP: at 10 mpf, when the CP had fully expanded in uncentrifuged control oocytes, the CP was still barely detectable in oocytes with displaced myoplasm (Fig. 6a and Supplementary Video 9). In the few cases where a CP became detectable, the CP seemed considerably smaller and wider than typically observed in control oocytes (Fig. 6a, arrowhead). Absence of the myoplasm from the vegetal cortex also affected the more global changes in cell shape found in non-centrifuged oocytes: upon fertilization, and different from control oocytes, centrifuged oocytes showed a shortening of the AV axis and no expansion of the VP, consistent with our finding that no CP forms in these oocytes (Fig. 6b,c). Notably, the actin cortex in centrifuged oocytes upon fertilization opened at the AP (Fig. 6a, asterisks) and accumulated at the VP (Extended Data Fig. 8d), displaying a flow profile that varied from oocyte to oocyte but overall resembled the cortical actin flow profile in control oocytes. Note that despite the seemingly normal cortical actin flows, oocyte shape changes associated with CP initiation were not clearly recognizable in centrifuged oocytes (Fig. 6b,c), likely because of the ectopic position of the myoplasm locally disrupting cortical actin flows. Collectively, these findings support the notion that myoplasm buckling by cortical actomyosin flows is required for CP formation.

Finally, we tested whether actin cortex relaxation and thus the coalescence of myoplasm buckles into a single buckle is required for CP formation. To this end, we treated unfertilized oocytes with 1 µM Ionomycin, leading to a slow and uniform increase in intracellular calcium^45,46^ (Fig. 6d–f). Treated oocytes displayed prolonged vegetal-directed cortical actin flows (Fig. 6e,f and Supplementary Video 10) and delayed cortex disassembly/relaxation at the VP, leading to continuous myoplasm buckling and impaired buckle coalescence (Fig. 6g,h). Collectively, this supports the notion that myoplasm buckle coalescence into a single buckle upon vegetal cortex relaxation drives CP formation.

Taken together, our data show that ooplasmic reorganization and CP formation in ascidian oocytes depend on the combined activities of the actomyosin cortex and the mitochondria-rich myoplasm through friction forces arising at their interface. Previous studies have indicated that fertilization-induced cortical actomyosin contraction, potentially mediated by a transient increase in intracellular Ca^2+^ levels^25^, directs CP formation^19,44,47,48^. Our data show that CP morphogenesis occurs in three distinct phases—CP initiation, CP expansion and CP absorption—and that cortical actomyosin contraction and flow seem to be primarily involved in CP initiation. Here, increased cortical tension along the AV axis of the oocyte upon fertilization leads to opening of the cortex at the AP, where actin density is lower. This again triggers vegetal-directed cortical actomyosin flows that polarize the cortex and deform the VP. CP expansion, in contrast, is not a direct consequence of these cortical flows, as previously indicated^17,47,48^, but is driven by friction forces arising at the interface between the more stationary myoplasm—which behaves as a solid-like layer similar to the Balbiani body of *Xenopus* and zebrafish oocytes^40^—and the directly adjacent flowing actomyosin cortex. These friction forces cause the myoplasm to buckle, with the shape of these buckles again deforming the VP and leading to CP expansion. Thus, CP formation being achieved by the partially overlapping functions of cortical actomyosin flow driving CP initiation and myoplasm buckling driving CP expansion might represent a mechanism conferring robustness to CP formation.

Friction forces arising between adjacent tissues have previously been shown to have an important morphogenetic function in tissue positioning during embryo development. In particular, friction forces generated at the interface between embryonic tissues undergoing active rearrangements and more stationary extracellular structures, such as the eggshell, were shown to modulate tissue morphogenesis^14,15^. Our data in ascidian oocytes show that friction forces play also a decisive morphogenetic role on a cellular and subcellular scale by influencing cytoplasmic rearrangements and cell shape changes. Intriguingly, friction forces predominantly function in these processes not only by modulating cortical actin flows, as typically assumed when incorporating friction to describe actin flows^9^, but also by compressing the subcortical myoplasm, which in turn triggers oocyte shape changes at the VP once cortical actin flows have ceased. This reveals a mechanism by which friction forces exert their morphogenetic function in development.

## Methods

### Animal maintenance and embryo handling

Adult *Phallusia mammillata* were purchased from Roscoff Marine Station (European Marine Biological Resource Centre) and kept in artificial sea water (ASW; Tropic Marin BIO-ACTIF Sea Salt, Tropic Marine) aquariums at 16 °C for 3–4 weeks under constant light. All embryo manipulations and experiments were carried out at 16 °C. Oocytes and sperm were obtained after animal dissection and kept at 4 °C for 3–4 days. Oocytes were chemically dechorionated in a 1% trypsin solution (Sigma-Aldrich, T8003) in ASW for 50 min under gentle stirring and kept in ASW supplemented with 0.05 g per l streptomycin sulfate salt (Sigma-Aldrich, S6501) until fertilization with activated sperm (resuspension of sperm in ASW, pH 9).

### Cloning of expression constructs

To generate the pSPE3-iMyo-mScarlet, Gibson assembly^49^ was used to create a new destination vector (pSPE3-R1-RfA-R3). The backbone for the new destination vector was amplified using the following primers: 5′-TATACATAGTTGGATAAACTTAGATATCGCGGCCG-3′ and 5′-GTCACTATGGTCGACCTGCAGAC-3′ from the vector pSPE3-RfA (a gift from Patrick Lemaire, University of Montpellier). The R3 arm of the new destination vector was amplified from pDEST-R4-R3 using the following primers: 5′-AGGTCGACCATAGTGACTGGATATGTTGTGTTTTACAGTATTATGT-3′ and 5′-ATCTAAGTTTATCCAACTATGTATAATAAAGTTGAACGAGAAACGTAAAATGA-3′. Polymerase chain reaction (PCR) fragments were assembled following the one-step Gibson assembly protocol.

The following primers with gateway arms were used to amplify the coding region for mScarlet from the vector Bra>iMyo-mScarlet (a gift from Edwin Munro, University of Chicago): 5′-GGGGACAGCTTTCTTGTACAAAGTGGCTATGGTGAGCAAGGGCGAGG-3′ and 5′-GGGGACAACTTTGTATAATAAAGTTGCTTACTTGTACAGCTCGTCCATGCCG-3′′. The PCR product was used to generate an entry vector using the pDONR-P2r-P3 (p3M-mScarlet). The following primers with gateway arms were used to amplify the coding region for iMyo from the vector Bra>iMyo-mScarlet: 5′-GGGGACCACTTTGTACAAGAAAGCTGGGTAACCTAGGACGGTCAGCTTGG-3′ and 5′-GGGGACAAGTTTGTACAAAAAACAGGCTTAATGGCCGAGGTGCAGCT-3′. The PCR product was used to generate an entry vector (p5M-iMyo) using the pDONR221. Entry vectors were further recombined with the destination vector pSPE3-R1-RfA-R3.

To generate the pSPE3-iMyo-mKO2 vector, the following primers with gateway arms were used to amplify the coding region for mKOkappa-2A from the vector mKOkappa-2A-mTurquoise2 (a gift from Dorus Gadella; Addgene plasmid no. 98837 (ref. ^50^)): 5′-GGGGACAGCTTTCTTGTACAAAGTGGTAGGCGAGGAGAGTGTGATTAAACC-3′ and 5′-GGGGACAACTTTGTATAATAAAGTTGTTCGCCAGTGGAATGAGCTACT-3′. The PCR product was used to generate a p3M-mKO2 entry vector via recombination with pDONR-P2rP3. p3M-mKO2, p5M-iMyo and pSPE3-R1-RfA-R3 were recombined to generate the expression construct.

To generate the pSPE3-Utrophin-mScarlet vector, the entry clones M5-pCR8-GW-TOPO-Utrophin (a gift from Edwin Munro, University of Chicago), p3M-mScarlet and pSPE3-R1-RfA-R3 were recombined to generate the expression construct.

To generate the pSPE3-iMyo-mNeonGreen vector, the entry clones p5M-iMyo, p3M-mNeonGreen (Heisenberg lab) and pSPE3-R1-RfA-R3 were recombined to generate the expression construct.

### mRNA microinjections

Microinjection needles (World Precision Instruments, 1B100-4) were pulled with a P-97 needle puller (Sutter Precision Instruments) and mounted on a microinjection setup (Narishige) attached to an Olympus SZX16 stereomicroscope. Dechorionated oocytes were placed in agarose wells. In vitro mRNA transcription was performed using the mMESSAGE mMACHINE T3 Transcription Kit (Ambion, AM1343). The following mRNAs were injected in dechorionated unfertilized oocytes: *Utrophin-Venus* (a gift from Alex McDougall, Sorbonne University), *Utrophin-mScarlet* (this study), *iMyo-YFP* (a gift from Edwin Munro, University of Chicago), *iMyo-mScarlet* (this study), *iMyo-mKO2* (this study), *Utrophin-mNeonGreen* (this study) and *CaMypt*^51^. mRNAs were injected at a concentration of 1–1.5 µg ml^−1^ the evening before the experiments, except for *CaMypt* mRNA, which was injected 3–4 h before the experiment.

### Calcium labelling

Dechorionated unfertilized oocytes were injected with Calcium Green (Invitrogen, C3713) to visualize calcium.

### Myoplasm labelling

Dechorionated unfertilized oocytes were incubated in 1 µM MitoTracker Deep Red FM (Invitrogen, M22426) in ASW for 5 min and washed before fertilization.

### Cytoskeletal inhibitors

For inhibition of F-actin and microtubule polymerization, unfertilized oocytes were incubated in 0.1% DMSO (control, Sigma-Aldrich, D8418), 1 µg ml^−1^ LatB (F-actin; Sigma-Aldrich, L5288), 0.6 µM Nocodazole (microtubules; Sigma-Aldrich, M1404) or 100 µM Colchicine (microtubules; Sigma-Aldrich, C9754) in ASW for 10 min before fertilization.

### Ionomycin treatment

Dechorionated unfertilized oocytes were incubated in 1 µM Ionomycin (Sigma-Aldrich, I3909) in ASW immediately before imaging.

### Confocal live imaging

For confocal life imaging, unfertilized oocytes were mounted in transparent microwells^20^. Briefly, in a MatTek dish (MatTek, P35G-1.5-14-C), a polydimethylsiloxane stamp with micropillars of different sizes was pressed onto a small drop of a polymer (MY Polymers, MY-134). The polymer/stamp was purged in nitrogen for 5 min, placed under 365 nm UV light (100 mW UV light-emitting diode M365L2-C1 from Thorlabs/Germany at about 1 mW cm^2^) for 15 min and cured for a further 1 h 45 min without nitrogen. Finally, the polydimethylsiloxane stamp was peeled out, and the microwells were coated with 1% gelatine from bovine skin (Sigma-Aldrich, G9391). Oocytes were imaged with an inverted Leica TCS SP5 confocal microscope equipped with a HC PL APO CS2 40x/1.10 NA water immersion objective (Leica) and resonant scanner. Temperature was kept at 16 °C using a custom-made cooling stage. The entire oocyte was imaged with an optical section thickness of 3 µm every 16–20 s.

### Micropipette aspiration experiments

Cortical tension measurements^52^ were performed using dechorionated oocytes placed in a gelatine-coated MatTek dish (MatTek, P50G-0-14-F) on an inverted Leica TCS SP5 microscope. Blunt, fire-polished and heat-inactivated fetal bovine serum-passivated (Fisher Scientific, 15961842) micropipettes with a 20 µm inner diameter (Biomedical Instruments) and a 30° bent angle were positioned by micromanipulators (TransferMan Nk2, Eppendorf) at the surface of the AP or VP of unfertilized oocytes. A negative pressure was applied with an increment of 10 Pa using a Microfluidic Flow Control System Pump (Fluiwell, Fluigent) and Dikeria micromanipulation software^53^. Images were acquired every second. The length of the deformation inside the pipette was measured in Fiji^54^ using a customized macro and plotted over time. Cortical (or surface) tension (*T*_c_) was calculated using the Young–Laplace equation:

$${T}_{\text{c}}=\frac{{P}_{\text{c}}}{2\left(\frac{1}{{R}_{\text{p}}}-\frac{1}{{R}_{\text{c}}}\right)}$$

*P*_c_ is the critical pressure reached when the deformation inside the pipette is equal to the pipette radius (*R*_p_); *R*_c_ is the radius of the oocyte at the initial aspiration time point. Controls were performed with pipettes of different sizes (10, 20, 35 and 40 µm) (Extended Data Fig. 5c).

### Cortical tension in fertilized oocytes

Cortical tension changes upon fertilization were monitored using micropipette aspiration (‘Micropipette aspiration experiments’). Micropipettes (blunt, 30° bent angle, passivated with heat-inactivated fetal bovine serum, inner diameter 40 µm) were placed at the AP or VP of unfertilized oocytes, and a negative pressure was applied until the deformation in the pipette reached the pipette radius (30 Pa and 45 Pa for AP and VP, respectively). The oocyte was then fertilized, and the aspirated pressure was modified so that the length of the deformation inside the pipette remained constant over time. The fertilization time point was identified by a transient elevation of intracellular calcium as detected by injection of Calcium Green.

### Viscosity measurement in unfertilized oocytes

For viscosity measurements^55^, unfertilized dechorionated oocytes were placed in a gelatin-coated MatTek dish (MatTek, P50G-0-14-F) on an inverted Leica TCS SP5. Blunt, fire-polished and passivated micropipettes with a 20 µm inner diameter were positioned at the surface of the oocyte. A negative pressure of 150 or 100 Pa (for control/DMSO and LatB-treated oocytes, respectively) was applied until the aspirated tongue flowed with a constant velocity into the pipette, followed by immediate pressure release. To calculate the viscosity, the length of the aspirated tongue was measured with a custom-made Fiji macro and plotted over time. Because the aspiration and retraction curves varied substantially in an experiment, only the aspiration curve was used for calculating viscosity, $\eta$, as follows:

$$\eta =\frac{{R}_{\text{p}}\left(P-{P}_{\text{c}}\right)}{3\uppi {L}_{{\text{asp}}}}$$

where *P* is the applied pressure, *P*_c_ is the critical pressure reached when the deformation inside the pipette is equal to the pipette radius (*R*_p_) and *L*_asp_ is the slope of the aspiration curve. Controls were performed with pipettes of different sizes (15, 20 and 60 µm) (Extended Data Fig. 6b).

### Elastic modulus calculation

The relaxation time, $\tau$, was obtained from the creep curve determined in the viscosity ($\eta$) aspiration experiments (‘Viscosity measurements in unfertilized oocytes’) and used to calculate the Young modulus^55^ *E* as follows:

$$\tau \approx \frac{\eta }{E}$$

### Myoplasm buckling assay

A blunt micropipette with a 30° bent angle and an inner diameter of 20 µm was placed at the lateral side or VP of an unfertilized oocyte with the myoplasm labelled. A negative pressure below the critical pressure was applied for 4–10 min, and the deformation of the myoplasm was monitored over time using an inverted Leica TCS SP5 confocal microscope.

### UV-laser ablation

UV-laser ablation of unfertilized oocytes was performed on an Axio Observer Z1 (Zeiss) inverted microscope equipped with a confocal spinning disk unit (Andor Revolution ImagingSystem, Yokogawa CSU-X1), a Q-switched solid-state 355 nm UV-A laser (Powerchip, Teem Photonics) and a C-APOCHROMAT ×40/1.2 W Korr UV-VIS-IR water immersion objective. Unfertilized oocytes were either injected with *Utrophin-Venus* mRNA one day before the experiment and/or incubated in MitoTracker Deep Red FM to label actin and/or myoplasm, respectively, and oriented with their AP (for actin) or VP (for myoplasm) facing the objective. The AP actin cortex was cut close to the meiotic spindle-associated actin patch along 3–4 15 µm lines by applying 25 UV pulses at 1 kHz. The oocyte was then imaged using an exposure time of 150 ms and a 0.2 s frame rate. Cortical retraction following ablation was measured from the maximum recoil of the animal-most cortex around the ablation site. A line was drawn perpendicular to the cut to generate a kymograph (using the KymoResliceWide v.0.5 plugin for Fiji; https://github.com/ekatrukha/KymoResliceWide) from which the maximum deformation was measured and the AP and VP flow velocities were calculated. The myoplasm was cut at the VP of the oocyte along a 10 µm line by applying 25 UV pulses at 1 kHz. The oocyte was then imaged using an exposure time of 150 ms and a 0.2 s frame rate. A line was drawn perpendicular to the cut to generate a kymograph (using the KymoResliceWide v.0.5 plugin for Fiji; https://github.com/ekatrukha/KymoResliceWide).

### AR and pole-displacement calculations

To calculate the AR of oocytes/zygotes, 10 brightfield z-slides over the equator of the cell were sum-projected and segmented using ilastik software^56^ and the pixel-classification method. The segmented images were stabilized (using the StackReg plugin in Fiji), and the CM was obtained for each time point. Because the CM oscillated between 1 and 4 pixels (0.33–1.2 µm on average) during the time-lapse (within the error margin), an average was taken. Using a custom-made Fiji plugin, the distance from the CM to the AP, the VP and the right- and left-most positions was measured over time. The AR was then calculated as the ratio of the sum of the animal and vegetal distances to the sum of the right and left distances.

### Actin, myosin and myoplasm fluorescence intensity quantifications

To determine the total fluorescence intensity of actin-, myosin II- and myoplasm-labelled oocytes, segmented images were used as regions of interest (ROIs) for measuring entire oocyte sum projections using the Analyze Particles option in Fiji. To measure the animal and vegetal intensities, only the animal or vegetal half of the oocyte, respectively, was measured. Total fluorescence intensities were batch-normalized by modelling the background fluorescence decay as an exponential (as implemented in Python: scipy.optimize.curve.fit (https://docs.scipy.org/doc/scipy/reference/generated/scipy.optimize.curve_fit.html)). To calculate the cortex to cytoplasm ratio of actin and myosin II fluorescence over time, sum projections of 10 confocal sections (15 µm) over the equator of the cell were segmented using ilastik software and the pixel-classification method to obtain the cortex and cytoplasmic regions. The segmented images were then used to obtain the cortical and cytoplasmic intensities over time.

### Temporal cross-correlation analysis

To determine whether actin and myosin II dynamics are in phase/correlated in time, the normalized actin and myosin II total intensities were time-delay correlated to evaluate whether a time shift between them exists^57^.

### Polar plots and curvature analysis

The images from the segmented actin cortices were stabilized (using the StackReg plugin in Fiji) and processed by means of a custom-made Python script to create the polar plots.

For curvature analysis, 10 brightfield z-slides over the equator of the cell were sum-projected and segmented using ilastik software and the pixel-classification method. The segmented images were stabilized (using StackReg plugin in Fiji) and processed using a custom-made Python script to calculate the curvature.

### Flow velocity analysis

To measure actin and myosin II flow velocities (in two dimensions) in oocytes, a 200 × 300-pixel ROI was selected in maximum intensity projections centred along its AV axis. To measure bulk cytoplasm flow velocities (in two dimensions), a 200 × 300-pixel ROI parallel to the AV axis (AV axis flow velocities) or perpendicular to it (left–right axis flow velocities) was selected in central brightfield slides of the entire oocyte.

The respective ROI was then used for PIV analysis using PIVlab^58^ and postprocessed by a custom-made MATLAB script as described^22^. Flow velocities represented in the kymographs were averaged over three consecutive time points.

### EM (sample preparation)

For high-pressure freezing, unfertilized oocytes were placed in 3 mm diameter aluminium carriers (Wohlwend) in 5% Bovine Serum Albumin (BSA; Sigma-Aldrich, A7906) in ASW and frozen using a HPM010F (BalTec). Freeze substitution was carried out using an AFS2 (Leica Microsystems) with an agitation module^59,60^. Samples were incubated in 0.1% tannic acid in acetone at −90 °C for 24 h, followed by 3 × 10 min washes in acetone at −90 °C. Samples were then placed in 2% osmium tetroxide, 0.1% uranyl acetate in acetone at −90 °C for 4 h before the temperature was raised at 15 °C per h steps to −60 °C, followed by incubation at this temperature for 3 h, a step-wise temperature rise of 15 °C per h to −30 °C and incubation for 3 h at this temperature. Afterwards, the temperature was raised at 15 °C per h steps to 4 °C, and the samples were incubated for 1 h at this temperature. Finally, samples were washed 3 × 10 min in acetone on ice, infiltrated in a graded series of hard Durcupan ACM resin in acetone (1:3, 1:1, 3:1), placed in pure resin overnight at room temperature and then transferred to BEEM capsules, filled with fresh resin and polymerized for three days at 60 °C.

### Transmission EM

Ultrathin sections (70 nm) were cut with a 4 mm Ultra 35° diamond knife (Diatome) using an ultramicrotome (EM UC7, Leica Microsystems) and mounted on Formvar-coated copper slot grids. Images were taken with a transmission electron microscope Tecnai 10 operated at 80 kV (FEI/Thermo Fisher Scientific) and processed in Fiji.

### Scanning EM

For serial sectioning, the blocks were trimmed with an Ultratrim diamond knife (Diatome) to a rectangle and a depth of 100 mm using an ultramicrotome (EM UC7, Leica Microsystems). Before sectioning, a carbon-coated Kapton tape (RMC Boeckeler) was plasma-treated using an ELMO glow discharge cleaning system (Agar Scientific) equipped with a homemade reel-to-reel motorized winder. Serial sections of 70 nm thickness were cut and picked up using an automated tape-collecting ultramicrotome (ATUMtome, RMC Boeckeler). After collecting the sections, the tape was mounted on 4-inch silicon wafers (University Wafer) with conductive double-sided adhesive carbon tape (Science Services). The wafers were then coated with a 5 nm carbon layer to ensure conductivity (EM ACE600, Leica Microsystems). Sections were imaged by a scanning electron microscope (FE-SEM Merlin compact VP, Carl Zeiss) equipped with the Atlas 5 Array Tomography software (Carl Zeiss). High-resolution serial images were taken with 5 nm-pixel resolution at 5 kV using a backscattered electron detector. The serial images were then concatenated, aligned and segmented using TrackEM (NIH). Segmented images were imported into Imaris software for visualization (Bitplane).

### Myoplasm length and buckling analysis

Analysis of the myoplasm buckles was performed using confocal microscopy images (central sections) of actin- and myoplasm-labelled oocytes. Images were binarized, and the signal was fitted to an ellipse^61^ in polar coordinates. Then a second fit of the surface modes was performed on the fitted ellipse with Gaussian basis functions using linear regression to extract the spatial coordinates of the myoplasm buckles as a function of their position along the vegetal cortex (centred at the VP). The length of the myoplasm and the underlying actin cortex was measured using the segmented-line function in Fiji.

### Centrifugation of unfertilized oocytes

To displace the myoplasm in unfertilized oocytes, oocyte centrifugation was performed^62^ by placing Utrophin-Venus-expressing unfertilized oocytes on a 5–30% Ficoll PM400 (Sigma-Aldrich, 26873-85-8) in ASW continuous gradient (Gradient Station, Biocomp) and centrifuged for 1 h at 1,500*g* at 16 °C in an Beckman Optima XPN-100 Ultracentrifuge (Beckman Coulter) using a swinging-bucket rotor (SW 41 Ti, Beckman Coulter). Centrifuged oocytes were collected and washed at least five times in ASW. Oocytes with the myoplasm displaced from the vegetal-most cortex were then incubated in MitoTracker Deep Red FM to label the myoplasm and fertilized under the microscope.

### Whole-mount immunofluorescence

Oocytes were fixed in 4% formaldehyde (Sigma-Aldrich, F8775) in ASW at 16 °C for 1 h under gentle stirring. After fixation, they were washed three times in PBS-Tw (0.1% Tween20 in PBS), permeabilized for 30 min in 0.1% Triton 100-X in PBS and washed three times in PBS-Tw. Oocytes were then incubated in blocking solution (0.1% Tween20, 0.5% BSA in PBS) for 30 min, followed by incubation in the primary antibody in PBS-BSA (1% BSA in PBS) for 24 h at room temperature and three washes in PBS-Tw. Incubation of the secondary antibody and phalloidin (1/200, Alexa FluorTM 488 Phalloidin, Invitrogen, A12379) was carried out for 24 h at room temperature. To label the maternal and paternal DNA, Hoechst (Fisher Scientific, 62249) was added at a final concentration of 5 µg ml^−1^ for the final 10 min of incubation. After incubation, oocytes were washed three times in PBS-Tw and one time in PBS and mounted for imaging. The primary antibodies were mouse anti-neurofilament (1/400, Sigma-Aldrich, N5264, for labelling the myoplasm), rabbit anti-phospho-S6 (1/400, Cell Signaling Technology, 2211, for labelling the endoplasmic reticulum) and rabbit anti-phospho-Myosin light chain (1/500, Cell Signaling Technology, 3674S, for labelling phospho-Myosin). The secondary antibodies were goat anti-mouse/rabbit conjugated to Alexa Fluor 488/546/647 (1/200) Molecular Probes).

### Data analysis and statistics

Statistical analysis was performed using Prism 8 (GraphPad). The statistical test used in each case and the resulting *P* values are indicated in each figure. *N* was considered as an independent experiment, and *n* denotes the number of oocytes; *N* and *n* are indicated in each figure. No statistical method was used to predetermine sample size. The experiments were not randomized.

### Reporting summary

Further information on the research design is available in the Nature Portfolio Reporting Summary linked to this article.

## Online content

Any methods, additional references, Nature Portfolio reporting summaries, source data, extended data, supplementary information, acknowledgements, peer review information; details of author contributions and competing interests; and statements of data and code availability are available at 10.1038/s41567-023-02302-1.

### Supplementary information

Supplementary Information Supplementary theory note.

Reporting Summary

Supplementary Video 1 Changes in cell shape upon fertilization of ascidian oocytes during CP formation, related to Fig. 1. Time-lapse brightfield of an exemplary ascidian oocyte during fertilization and CP formation. Scale bar, 30 µm. Time interval, 16 s.

Supplementary Video 2 Actomyosin cytoskeleton is required for CP formation, related to Fig. 1 and Supplementary Fig. 1. Time-lapse brightfield of exemplary ascidian oocytes treated with, from left to right and top to bottom: 0.1% DMSO, 1 µg ml^−1^ LatB, CaMypt overexpression and 0.6 µM Nocodazole during fertilization and CP formation. Scale bar, 30 µm. Time interval, 16 s.

Supplementary Video 3 Actomyosin dynamics during CP formation, related to Fig. 2 and Supplementary Fig. 2. Time-lapse fluorescence confocal video of central sections of an exemplary ascidian oocyte co-injected with Utrophin-mNeonGreen mRNA (green, left panel) and iMyo-mScarlet mRNA (magenta, right panel) to mark actin and myosin II, respectively. Scale bar, 30 µm. Time interval, 16 s.

Supplementary Video 4 Actin dynamics during CP formation, related to Fig. 2. Time-lapse fluorescence confocal video (maximum intensity projections) of an exemplary ascidian oocyte injected with Utrophin-Venus mRNA to mark actin. Scale bar, 30 µm. Time interval, 16 s.

Supplementary Video 5 Surface tension along the AV axis of unfertilized oocytes, related to Fig. 3 and Supplementary Fig. 4. Time-lapse fluorescence confocal video (maximum intensity projections) of an exemplary ascidian oocyte injected with Utrophin-Venus mRNA to mark actin. White line denotes the ablation site along the bright actin patch at the AP. Scale bar, 30 µm. Time interval, 8.2 s.

Supplementary Video 6 The myoplasm displays solid-like properties, related to Fig. 4. Time-lapse fluorescence video of the VP of an exemplary ascidian oocyte with the myoplasm labelled by MitoTracker Deep Red. White line denotes the ablation site. Scale bar, 15 µm. Time interval, 0.2 s.

Supplementary Video 7 The myoplasm buckles at the VP during CP formation, related to Fig. 5. Time-lapse fluorescence confocal video of central sections of an exemplary ascidian oocyte injected with Utrophin-Venus mRNA to mark actin (green, left panel) and treated with MitoTracker Deep Red (blue, right panel) to mark the myoplasm. Scale bar, 30 µm. Time interval, 16 s.

Supplementary Video 8 The myoplasm buckles under compression from the actomyosin cortex, related to Fig. 5. Time-lapse fluorescence confocal video of an exemplary unfertilized ascidian oocyte with the myoplasm labelled by MitoTracker Deep Red. White lines denote the sides of the pipettes positioned at the VP of the oocyte. Scale bar, 30 µm. Time interval, 1 s.

Supplementary Video 9 Centrifuged oocytes do not form a CP, related to Fig. 6. Time-lapse brightfield (top left panel) and fluorescence confocal video of an exemplary ascidian oocyte injected with Utrophin-Venus mRNA to mark actin (green, top right panel; maximum intensity projections) and treated with MitoTracker Deep Red to label the myoplasm (blue, bottom panels; right, central section; left, maximum intensity projections). Scale bar, 30 µm. Time interval, 16 s.

Supplementary Video 10 Ionomycin-treated unfertilized oocytes display continuous myoplasm buckling and do not form a CP, related to Fig. 6. Time-lapse fluorescence confocal video of an exemplary unfertilized ascidian oocyte treated with 1 µM Ionomycin and injected with Utrophin-Venus mRNA to mark actin (green, left panel; sum intensity projections) and treated with MitoTracker Deep Red (blue, right panel; central sections) to mark the myoplasm. Scale bar, 30 µm. Time interval, 16 s.

Supplementary Code 1 Code used for the theoretical model.

### Source data

Source Data Source data and statistical data for Figs. 1, 3, 4, 5 and 6 and Extended Data Figs. 1, 2, 3, 5, 6, 7, 8.

## Data Availability

Source data are provided with this paper. All other data supporting the findings of this study are available from the corresponding author on request.

## Code availability

Code is available in the supplementary file Ascidian_oocyte_model.nb.
