# Supplementary material for: Friction forces determine cytoplasmic reorganization and shape changes of ascidian oocytes upon fertilization
Source: Nat Phys. 2024 Jan 9;20(2):310–21. doi: 10.1038/s41567-023-02302-1 (PMC10866705; doi:10.1038/s41567-023-02302-1)
Supplement: Supplementary file 1 — Supplementary theory note. [file 41567_2023_2302_MOESM1_ESM.pdf]

nature physics

Article

https://doi.org/10.1038/s41567-023-02302-1

Friction forces determine cytoplasmic reorganization and shape changes of ascidian oocytes upon fertilization

In the format provided by the authors and unedited

Supplementary Theory Note

I. OVERVIEW OF MODEL ASSUMPTIONS

We present in this section a theoretical model that aims at describing the dynamics of cortical flows and shape changes of an ascidian oocyte following fertilization. Before fertilization, we assume that the cell is a sphere of radius R, with a uniform actomyosin cortex with (two-dimensional) density ρ_0. Following experimental findings, we model the effect of fertilization (occurring at time $t = 0$) by an abrupt drop in cortical density in the neighborhood of the animal pole (AP), denoted by the polar angle $\theta = \pi$ (see Figure S3D), and a global increase of contractility that drives the cortex to an unstable state, as will be explained below.

Experimentally, fertilization at the AP causes an initial cortex retraction and cell elongation (Figures 1 and 2 of the main text, $0 \lesssim t \lesssim 1$ min 30 s). At later times, a moving cortical density front arises [Figure 2A (red arrowhead), 1 min $\lesssim t \lesssim 3$ min 30 s], appearing more markedly near the equator line of the cell, and traveling towards the vegetal pole (VP, at $\theta = 0$); the position of the front coincides with a "pinching" of the cell near the VP, and results in its bulging shape. Note that the front, in its early stages upon fertilization near the AP ($0 \lesssim t \lesssim 1$ min 30 s), seems to be distinct from the increase in cortex density observed near the equator line, until they merge. The relative increase of cortex density between the equator and the VP correlates with the underlying myoplasm, which is most pronounced between the equator and the VP ; this structural heterogeneity of the cortex is not explicitly described in the model.

Below we relate the tensions arising in the cortex after fertilization to the observed onset of cortical flows and sequence of shape changes. In the model, we assume that the deformed cell is axisymmetric around the AP-VP, or z, axis. We will assume small departures from the initial spherical shape, that is, the surface displacement $|e(\theta)| \ll R$ (see Figure S3D). Under this assumption, we perform a perturbative calculation whereby the cortical tensions are first obtained from a tangential force balance on a sphere. The cortical tensions are then inputs in a calculation of the shape change, $e(\theta)$, determined from Laplace's law. Last, we argue that friction forces between the flowing cortex and the myoplasm can lead to the buckling of the myoplasm, described as an elastic shell, which relaxes at later times. Overall, our model shows that a mechanical perturbation (as is triggered by fertilization) induces a massive dynamic reorganization of the actomyosin cortex and underlying myoplasm, which results in a non trivial sequence of cell shape changes leading to the formation of the cell contractile pole ; despite its relative simplicity, and purely physical origin, the model finally recapitulates most of experimental observations.

II. CORTICAL TENSION IN THE INITIAL STATE

Before fertilization, the total cell surface tension is assumed to be isotropic in the plane and given by

$$T_0 = T_\mathrm{m} + T_c \,, \tag{1}$$

where T_m is a contribution coming from the membrane plus cortex/membrane attachments and T_c is a purely cortical contribution. We assume some phenomenological dependence of $T_c(\rho)$ on cortical actin density ρ, given by

$$T_c(\rho) = -a\,\rho + \zeta\,\rho^2 - b\,\rho^3 \,. \tag{2}$$

This phenomenological functional form is a convenient, but not unique, choice that allows for bistability between low and high cortical densities, which is consistent with standard models of cortical dynamics [1–3]. In this equation a, ζ, and b are positive constants. The term with coefficient a is a phenomenological coefficient that effectively describes

repulsive interactions between filaments and stabilizes low densities; the second term, with coefficient ζ, represents the effect of contractility and can induce instabilities at intermediate densities; and finally the last term, with coefficient b, describes repulsive interactions and stabilizes high cortical density. Because the spherical shape is stable before fertilization, we take the values of the constants a, ζ, b, and the initial density ρ_0 such that the cortex is initially under tension, i.e., $T_c(\rho_0) > 0$; see Section VI for discussion of our choice of parameters. For a positive initial tension, the initial density satisfies

$$\rho_- < \rho_0 < \rho_+ \,, \tag{3}$$

where

$$\rho_\pm = \frac{\zeta}{2b}\left(1 \pm \sqrt{1 - \frac{4ab}{\zeta^2}}\right) . \tag{4}$$

In between $\rho_\pm$, $T_c(\rho)$ has a maximum at

$$\rho_m = \frac{\zeta}{3b}\left(1 + \sqrt{1 - \frac{3ab}{\zeta^2}}\right) . \tag{5}$$

The cortex is initially stable if $dT_c/d\rho|_{\rho=\rho_0} < 0$, otherwise it is unstable. To account for the cell-scale flow and accumulation observed in experiment, we assume that fertilization renders the cortex unstable (eg by increasing ζ), and locally depletes the cortex at the AP. For this to be the case, we assume that $\rho_- < \rho_0 < \rho_m$, and that $\zeta > \sqrt{3ab}$ in order for the maximum to exist.

III. CORTICAL DENSITY, FLOW, AND TENSIONS AFTER FERTILIZATION

Upon fertilization, the cortex retracts and the density is inhomogeneous. Assuming axisymmetry about the z-axis, we denote the cortical density on the undeformed sphere to be $\rho(\theta, t)$ and the flow velocity $\mathbf{v} = v(\theta, t)\,\mathbf{e}_\theta$, where $\mathbf{e}_\theta$ is the unit tangent vector to the sphere in the direction of increasing θ (see Figure S3). The tensions along the polar and azimuthal directions are

$$T_\theta(\theta, t) = T_\mathrm{m} + T_c(\rho) + \gamma\nabla^2\rho + 2\eta(\nabla\mathbf{v})_{\theta\theta} \tag{6}$$

$$T_\phi(\theta, t) = T_\mathrm{m} + T_c(\rho) + \gamma\nabla^2\rho + 2\eta(\nabla\mathbf{v})_{\phi\phi} \,. \tag{7}$$

In the equations for T_θ and T_ϕ the first three terms on the right constitute the isotropic contributions; the contribution $T_\mathrm{m} + T_c(\rho)$ has been introduced above in Equation 1 ; the third term, with coefficient $\gamma > 0$, penalizes abrupt changes in density and comes from a term $\gamma/2(\nabla\rho)^2$ in the cortex free energy density. The last terms are the anisotropic viscous contributions, with viscosity $\eta > 0$. Note that on a sphere the Laplacian is $\nabla^2 = R^{-2}\frac{1}{\sin\theta}\partial_\theta(\sin\theta\partial_\theta)$ and the strain rate components are $(\nabla\mathbf{v})_{\theta\theta} = R^{-1}\partial_\theta v$ and $(\nabla\mathbf{v})_{\phi\phi} = R^{-1}\cot\theta\, v$.

Ignoring inertia, the tensions must satisfy force balance. Local balance of forces in the θ direction is expressed by

$$\partial_\theta T_\theta + \cot\theta(T_\theta - T_\phi) = \xi\, R\, v \,. \tag{8}$$

In the above, ξ is an effective friction coefficient that accounts for the interactions between the cortex and the cell bulk, ie the cytoplasm and the myoplasm. We note that, in general, it could be θ-dependent, but since including an angle dependence did not qualitatively change the results given below, we assumed that ξ is constant ; we will argue that in the experimentally relevant regime, friction forces with the myoplasm will remain small as compared to viscous forces.

Since cortical mass is conserved, ρ and v are also linked by the mass conservation equation:

$$\partial_t\rho + \nabla\cdot(\rho\mathbf{v}) = -k_d(\rho - \rho_0) \,. \tag{9}$$

In the above the divergence operator on the sphere is $\nabla \cdot () = \frac{1}{R \sin\theta} \partial_\theta [\sin\theta ()]$. The right-hand side above reflects turnover, and k_d is the cortical depolymerization rate.

Equations 8-9 are solved subject to the following boundary conditions:

- $\partial_\theta \rho(\theta = 0) = \partial_\theta \rho(\theta = \pi) = 0$. This is a consequence of axisymmetry and regularity of $\rho(\theta, t)$;
- $v(\theta = 0) = v(\theta = \pi) = 0$. This is a consequence of regularity of $v(\theta, t)$.

We also specify initial conditions for ρ and v:

- We set $v(\theta, t = 0) = 0$ since there is no flow initially.
- We set $\rho(\theta, t = 0) = \rho_0$ everywhere except close to $\theta = \pi$ where $\rho = \rho_{\text{edge}} < \rho_0$. As discussed above, we choose ρ_0 and/or the extent over which $\rho = \rho_{\text{edge}}$ so that the cortex is initially unstable. (If, in contrast, we were to assume that the cortex is initially stable, then the perturbation to the cortex given by the initial condition would relax and the cortical density would tend to a constant value given by ρ_+. Experimental observations rule out this possibility.)

Equations 8-9 are then solved, obtaining ρ and v, as shown in Figure 3 of the main text. We notice that at long times the density near $\theta = 0$ (VP) tends to the stable fixed point $\rho = \rho_+$, while near $\theta = \pi$ (AP) it tends to the stable fixed point $\rho = 0$. The flow that remains at steady state is due to a small but finite turnover rate k_d, used here for computational reasons.

Once the density and flow profiles are determined, the cortical tensions can be calculated from Equations 6 and 7. In particular, the model makes a prediction for how the total tension, $T = (T_\theta + T_\phi)/2$, at the VP ($\theta = 0$) and AP ($\theta = \pi$) evolve in time; see Figure 3 of the main text. The overall trend is consistent with our measurements using micropipette aspiration, showing good agreement with the model.

IV. SHAPE CHANGE

The oocyte undergoes dramatic shape changes in the first few minutes post-fertilization. We therefore sought to find out if our model could account for these deformations. To do so, we applied a perturbative approach whereby the cortical tensions $T_\theta(\theta, t)$ and $T_\phi(\phi, t)$ were calculated on the spherical reference surface, which were then used as input to find the deformed shape to first order in the surface displacement $e(\theta, t)$. We assumed that the cell is axi-symmetric around the AV axis, and we used Laplace's law to relate the cell shape to tension in order to find $e(\theta, t)$. This law tells us that the normal stress exerted by the cytoplasm on the cortex at a given position is related to the local cortical tensions and principal curvatures:

$$\sigma_{nn} = -K_\theta T_\theta - K_\phi T_\phi \,, \tag{10}$$

where σ_{nn} is the normal stress and K_θ and K_ϕ are the meridional and azimuthal curvatures of the cortex. To leading order in e, they are

$$K_\theta = \frac{1}{R}\left(1 - \frac{e}{R} - \frac{e''}{R}\right) \tag{11}$$

$$K_\phi = \frac{1}{R}\left(1 - \frac{e + e' \cot\theta}{R}\right) \,, \tag{12}$$

where the primes refer to derivatives with respect to θ. As a result, the leading order Laplace law is

$$\delta p = \frac{1}{R}(\delta T_\theta + \delta T_\phi) - \frac{T_0(\rho_0)}{R^2}\left(e'' + e' \cot\theta + 2e\right) . \tag{13}$$

In this equation, we have written $T_\theta = T_0(\rho_0) + \delta T_\theta$ and $T_\phi = T_0(\rho_0) + \delta T_\phi$, where δT_θ and δT_ϕ are assumed to be small quantities. Also, we have taken $\sigma_{nn} \simeq -p_0 - \delta p$. Here, p_0 is the initial pressure in the spherical model oocyte

prior to instability and δp is assumed small. The above can be written as an ordinary differential equation (ODE) for the surface displacement to be solved at a time t:

$$e'' + \cot\theta e' + 2e = \frac{R}{T_0(\rho_0)} \left(\delta T_\theta + \delta T_\phi - R\delta p\right) . \tag{14}$$

With δT_θ and δT_ϕ determined previously (Section III), this equation is a linear inhomogeneous ODE for e, giving the perturbed shape.

To solve equation 14 we must first determine the pressure perturbation δp, which can be done using two conservation laws. First, we assume constant oocyte volume, expressed as

$$\int_0^\pi e \sin\theta d\theta = 0 \,, \tag{15}$$

valid to linear order in e. Second, we neglect momentum exchange between oocyte and its surroundings, implying that the total force of the oocyte on the cortex projected along the symmetry axis vanishes:

$$F_z = \int_0^\pi (\xi v \sin\theta + \delta p \cos\theta) \sin\theta d\theta = 0 \,. \tag{16}$$

Next, we expand $\delta p(\theta, t)$ in terms of Legendre polynomials: $\delta p(\theta, t) = \sum_{l=0} \delta p_l(t) P_l(\cos\theta)$. Using equations 14 and 15 we first obtain $\delta p_0 = \frac{1}{2R}\int_0^\pi (\delta T_\theta + \delta T_\phi)\sin\theta d\theta$. From equation 16 we then obtain $\delta p_1 = -\frac{3}{2}\int_0^\pi \xi v \sin^2\theta d\theta$. Given that $v < 0$ — flow is directed from the AP to the VP — it follows that $\delta p_1 > 0$. Finally, we are free to truncate the expansion at $l = 1$, since at this level of perturbation theory the influence of higher modes on e can be assumed to come from the tensions δT_θ and δT_ϕ.

Thus, with δp determined, we obtain the perturbed shape by solving 14, subject to boundary conditions $e(\theta = 0) = 0$ (allowed by translation invariance of the shape) and $e'(\theta = 0) = 0$ (required by axi-symmetry). The resulting shape profiles are shown in Figure S3. Of note, this analysis shows that a non-vanishing friction ξ implies a non-uniform δp, with higher pressure at the VP than at the AP. This, in turn, indicates a non-trivial expression of Laplace's law, with total cortical tension over radius of curvature greater at the VP than at the AP, in agreement with experimental imaging and tension measurements. We finally note that δp can be determined explicitly from force balance, independently of any hypothesis on the rheology of the cell bulk. In particular, cytoplasmic flows might result from δp, but their analysis is not needed to determine it. In turn, the non uniform pressure non-trivially impacts on cell shapes as expressed by Laplace law, Eq. 14. This highlights the role of friction forces in the definition of cell shapes.

V. MYOPLASM BUCKLING

A. Friction as origin of compressive stress inside myoplasm

We model the myoplasm as a shallow elastic shell with a bending modulus B and radius R in contact with the cortex, as suggested by our experimental observations (Fig. 4A and B). The shallowness assumption is justified by noting that buckling occurs near the VP and on a length scale small compared with the cell radius. To further simplify, we first disregard the initial curvature of the myoplasm, reducing its initial shape to that of a disk, and return later to an account of its initial curvature. The external, frictional force density on the myoplasm is assumed proportional to the local actin cortical velocity, which is a non-monotonic function of position with a peak somewhere between cell equator and the VP; see Fig. 3D'. To simplify the model, and to obtain physical insight, we assume an effective, delta-function tangent force density at a position $r = a$, with $r = 0$ coinciding with the VP and $r = R$ the myoplasm edge:

$$\mathbf{f}_{\rm fr} = -\xi a V \delta(r - a)\,\hat{\mathbf{r}} \,, \tag{17}$$

where ξ is assumed to be the same friction coefficient as introduced earlier. We note that $V > 0$ is related to the amplitude of cortical flow; both a and V can in principle depend on time t. The position a should correspond to the peak of the cortical flow amplitude. The friction force induces compressive forces through the in-plane force balance along the r-direction:

$$\partial_r t_r + \frac{1}{r}(t_r - t_\phi) = \xi a V \delta(r-a)\,, \tag{18}$$

with t_r and t_ϕ the myoplasm, thickness-integrated stresses along the radial and azimuthal directions. We refer to these henceforth as myoplasm tensions, though they may be positive or negative. Assuming linear elasticity for the myoplasm the tensions are related to the two-dimensional strains u_r and u_ϕ as

$$t_r = \frac{Eh}{1-\nu^2}\left(u_r + \nu u_\phi\right) \tag{19}$$

$$t_\phi = \frac{Eh}{1-\nu^2}\left(u_\phi + \nu u_r\right)\,, \tag{20}$$

where E, h, and ν are the myoplasm Young's modulus, thickness, and Poisson ratio, respectively. Denoting the in-plane component of the displacement field as $u(r,t)$, the strains are $u_r = \partial_r u$ and $u_\phi = u/r$. Substituting these expressions into 20, then integrating 18 to give u, yields the following expressions for the tensions:

$$t_r = \begin{cases} -\dfrac{\xi a V(1+\nu)}{2}\left(1 + \dfrac{1-\nu}{1+\nu}\dfrac{a^2}{R^2}\right) & r < a \\ \dfrac{\xi a V(1-\nu)a^2}{2}\left(\dfrac{1}{r^2} - \dfrac{1}{R^2}\right) & r > a \end{cases}\,, \qquad t_\phi = \begin{cases} -\xi a V\dfrac{1+\nu}{2}\left(1 + \dfrac{1-\nu}{1+\nu}\dfrac{a^2}{R^2}\right) & r < a \\ -\dfrac{\xi a V a^2(1-\nu)}{2}\left(\dfrac{1}{r^2} + \dfrac{1}{R^2}\right) & r > a \end{cases}\,. \tag{21}$$

Note that the tensions are isotropic, uniform, and negative inside $r = a$; they are positive, non-uniform and anisotropic outside, because of the free edge condition $t_r = 0$ at $r = R$. The compressive tensions for $r < a$ can give rise to buckling, as we show in the next subsection.

B. Linear buckling analysis

To see how friction force exerted on the myoplasm gives rise to buckling, we write the displacement field as $\mathbf{u} = u\hat{\mathbf{r}} - w\mathbf{e}_z$, with $u(r,t)$ calculated previously and $w(r,t)$ the deflection due to buckling, assumed to be small. In the presence of bending moments, the force balance along z is

$$-\Gamma \partial_t w = B\nabla^4 w + t_r \kappa_r + t_\phi \kappa_\phi + Kw\,. \tag{22}$$

In this equation $-\Gamma$ is a dissipative coefficient, $B = Eh^3/12(1-\nu^2)$ is the myoplasm bending rigidity, and κ_r and κ_ϕ are the principal curvatures. The restoring force density Kw derives from an assumed harmonic energy penalty arising from deformation of the myoplasm's surroundings. This could, for instance, come from cytoplasmic elements linked to the myoplasm, such as cortical endoplasmic reticulum [5]. From dimensional analysis we expect that $K \sim E_{\rm cyto}/R$, with $E_{\rm cyto}$ the short-time elastic modulus of cytoplasm.

By substituting the tensions from equations 21 into 22 and focusing on the threshold of buckling (i.e., $\partial_t w = 0$), we obtain

$$\begin{aligned} &B\nabla^4 w + f\nabla^2 w + Kw = 0\,, \quad r \le a\,; \\ &B\nabla^4 w + (f - \Delta f)\left[\nabla^2 w - \frac{R^2}{r^2}\left(\partial_r^2 w - \frac{\partial_r w}{r}\right)\right] + Kw = 0\,, \quad r > a\,; \end{aligned} \tag{23}$$

where the force densities f and Δf are

$$f = a\xi V\frac{1+\nu}{2}\left(1 + \frac{1-\nu}{1+\nu}\frac{a^2}{R^2}\right) \tag{24}$$

$$\Delta f = a\xi V\frac{1+\nu}{2}\,. \tag{25}$$

Note that in the above we have used the small w expressions for curvature, namely $\kappa_r = -\partial_r^2 w$ and $\kappa_\phi = -\partial_r w/r$. To find the buckling threshold $f_{\rm cr}$, we look to solve equation 22 with the boundary condition that w and its derivatives vanish as r tends to R, assumed much greater than a. As there is no analytical solution for $r > a$ (at least to our knowledge), we use an approximate method based on matched asymptotics.

In applying this method we first note that the differential equation for w for $r \leq a$ — referred to as the interior region — can be solved, yielding

$$w_< = J_0(\mu_+ r/\lambda_B) + A J_0(\mu_- r/\lambda_B)\,, \tag{26}$$

where $J_0(z)$ is the Bessel function of the first kind of order zero; $\lambda_B = (B/K)^{1/4}$ is the characteristic length; and $\mu_\pm$ are positive solutions to

$$\mu^2 + \mu^{-2} = \frac{f}{\sqrt{BK}}\,. \tag{27}$$

Note that $\mu \sim n/(a/\lambda_B)$ is the re-scaled mode number, with n the number of nodes inside $r = a$; see inset of Fig. 5E.

There is, as mentioned before, no analytical solution for $r > a$, referred to as the exterior region. To find an approximate solution we rescale r by the length $\lambda = \sqrt{(f - \Delta f)/K} = \frac{a}{R}\sqrt{\frac{\xi V a(1-\nu)}{2K}}$. Doing so, the last two terms in equation 23b become comparable and with coefficient one, whereas the first term in the equation is of relative order $\epsilon \equiv (\lambda_B/\lambda)^4$. Anticipating that the threshold for buckling is $\xi \sim \sqrt{BK}/Va$ (see below), it follows that ϵ is of the order of one. Normally matched asymptotics is effective when the parameter in front of the highest derivative of the differential equation in question is small, and so it might seem questionable to use this method in our case. However, in the exterior region all three terms in equation 23 tend, for r greater than a, to dampen deflections w. We will see that this approximation gives good semi-quantitative results.

We separate the exterior region into two sub-regions: an "inner" one near $r = a$, where only the first two terms in equation 23 are kept; and an "outer" one where only the last two are kept. The reasoning behind this choice is that near $r = a$, where w changes rapidly, the derivative terms in the differential equation dominate; while in the far-field the highest derivative term is subdominant. A uniformly valid approximate solution, $w_>$, is obtained by matching the inner and outer solutions over a region $r - a \ll \lambda$ and $r - a \gg \sqrt{\epsilon}\lambda$. This leads to

$$w_>(r) \simeq C\, I_0\left(\frac{\sqrt{R^2 - r^2}}{\lambda}\right) + D \exp\left(\frac{r - a}{\lambda\sqrt{\epsilon}}\frac{R}{a}\right)\,, \tag{28}$$

where C and D are arbitrary constants and $I_0(z)$ a modified Bessel function of order zero. By enforcing continuity of w and its first three derivatives at $r = a$, from equations 26 and 28 the unknowns A, C, D, and the threshold for buckling, $f_{\rm cr}$, can be determined. The resulting solution for $w(r)$ at threshold is shown in Fig. 5E. As seen in the figure, the buckling wavelength near threshold is of order λ_B. Furthermore, there are roughly a/λ_B wavelengths that fit inside $r < a$. Thus, we see that the linear analysis predicts short wavelength buckling and recapitulates the experimental observation of myoplasm wrinkling near the end of contractile pole initiation (Figs. 5A and S6B, at 3mpf).

Finally, to verify the soundness of the asymptotic method used above we tested it with a one-dimensional problem, whose solution can be found exactly. A beam of length $2L$, lying between $x = -L$ and $x = L$, is subjected to delta-function compressive forces $-f\delta(x - a)$ and $+f\delta(x + a)$, with $a < L$. To correspond with the disk case, where $t_r > 0$ for $r > a$, we assume there is a constant tension T along the beam for $|x| > a$. We consider symmetric buckling and consider the beam for $x > 0$. The normal force balance equations near threshold read

$$B\partial_x^4 w + f\partial_x^2 w + Kw = 0\,, x < a\,; \tag{29}$$

$$B\partial_x^4 w - T\partial_x^2 w + Kw = 0\,, x > a\,. \tag{30}$$

These equations can be easily solved, yielding the critical buckling force $f_{\rm cr}$ and the deflection $w(x)$. For comparison, we may also use an asymptotic approach, nominally valid for $\epsilon = \lambda_B^4/\lambda_T^4 \ll 1$, where $\lambda_T = \sqrt{T/K}$. The interior solution, for $x < a$, is given by trigonometric functions,

$$w_<(x) = \cos(\mu_+ x/\lambda_B) + A\cos(\mu_- x/\lambda_B)\,, \tag{31}$$

where $\mu_\pm$ are solutions to equation 27. For the exterior solution, $x > a$, we obtain by asymptotic matching and to lowest order in ϵ,

$$w_>(x) = C \exp\left(\frac{-x}{\lambda_T}\right) + D \exp\left[\frac{-(x-a)}{\lambda_T\sqrt{\epsilon}}\right] . \tag{32}$$

As in the two-dimensional case, the four unknowns, A, C, D, and $f_{\rm cr}$ are found by continuity conditions of $w(x)$ and its first three derivatives at $x = a$. A comparison of the exact and the asymptotic solutions was done and the agreement is very good (not shown). We note that, even for ϵ of order one or larger, that the asymptotic approach gives good semi-quantitative results.

To conclude this discussion, we note that the finite buckling wavelength observed experimentally and predicted in our model crucially depends on the coupling to an elastic cytoplasm. The wavelength is set by the characteristic length scale $\lambda_B \equiv (B/K)^{1/4}$, similar to classical Euler buckling of a beam embedded in an elastic medium [7].

C. Myoplasm as a shallow shell

Lastly, we address the simplification made at the beginning of this section of reducing the myoplasm shell buckling to that of a flat disk. Noting that it occurs over a length scale λ_B that is small compared with the shell radius R, we can treat the myoplasm using shallow shell theory [6]. This accounts for two main corrections to the flat disk case: 1) the shell curvatures κ_r and κ_θ acquire terms of order $1/R$, independent of w; and 2) a uniform deflection w causes in-plane strains (equal to w/R) and hence modifies the tensions t_r and t_ϕ. As a result, to leading order in $1/R$ the in-plane force balance, equation 18 is unchanged, but the out-of-plane force balance, equation 22, becomes inhomogeneous and the elastic constant K is renormalized as

$$K \to K + \frac{E\,h}{R^2} . \tag{33}$$

Taking the elastic stiffness associated with cytoplasm deformation to be $\sim E_{\rm cyto}/R$ we see that the shell curvature-correction is of relative order $(Eh/E_{\rm cyto}R)$. Noting that $E \sim 1$ Pa and $E_{\rm cyto} \sim 1$ Pa, it is clear that this correction is small, justifying our flat disk approach to myoplasm buckling.

VI. CHOICE OF PARAMETER VALUES

A. Cortical flow - cell shape model

The flow-shape model described in Sections I through IV depends on a handful of parameters: R, ξ, η, $T_{\rm m}$, k_d, ρ_0, a, ζ, and b. We justify here our choices of parameters. The oocyte radius has been measured to be $R = 140$ µm. For simplicity we assume the membrane tension, $T_{\rm m}$, is zero, and the actin turnover rate is small: $k_d = 0.001$ min^{-1}, which is included for numerical stability and does not impact the results.

For the phenomenological parameters relating to the density-dependent tension T_c, we take ρ to be dimensionless, and assume without loss of generality that the initial value is $\rho_0 = 1$. The remaining three parameters are chosen based on measurements of cortical tension just after fertilization (~ 500 pN/µm), the maximum value at the VP (~ 1500 pN/µm), and the amplitude of cortical flow (~ 15 µm/min). In order for the orders of magnitudes determined from our model to match with these values, we chose $a = 1000$ pN/µm, $\zeta = 1700$ pN/µm, and $b = 467$ pN/µm. Finally, noting that the interface between the bright and dark regions of the cortex is sharp (Figure 2A of the main text), we assume the correlation length $\sqrt{\gamma/a}$ is of the order of 1 µm, which gives a value for the gradient penalty γ.

The (3D) actin viscosity has been measured here to be of the order of 10^3 Pa.s. We note, however, that a value of 10^5 Pa.s has been cited elsewhere [4]. Since cortex viscosity has a significant effect on cortical flow amplitude, to align our model with measurements we have taken $\eta_{\rm 3D} = 10^4$ Pa.s. Combining this with our measured value for the cortex thickness of $h = 2.5$ µm, the 2D viscosity we use is $\eta = 2.5 \times 10^{-2}$ Pa.s.m.

The friction coefficient ξ has been introduced in Eq. 8. In the force balance there are therefore two terms proportional to v, and their ratio defines the so-called hydrodynamic length

$$\ell = \sqrt{\frac{2\eta}{\xi}}\,. \tag{34}$$

Experiments suggests that ℓ is of the order or greater than the initial oocyte radius, R. Indeed, for this range of values of ℓ, flows are well-spread out between the AP and the VP, with a maximum shifted towards the VP. This model prediction is verified experimentally; see Fig. 3D (at 3mpf) and D'. In contrast, for ℓ smaller than R, i.e., higher friction, the theoretical flow curve decays rapidly (data not shown) as one approaches the VP, at odds with observation. For even smaller ℓ the friction becomes so strong that flow is only concentrated where the cortical density gradient is steepest (not shown). This gives rise to a local accumulation of material, and the instability mode shifts to a higher number (see [1]).

The effect of friction can also be clearly seen by comparing the total tension, $T = T_\theta + T_\phi$, at the VP ($\theta = 0$) and at the AP ($\theta = \pi$). Experimentally, T_{VP} is always higher than T_{AP}, and both increase sharply with time after fertilization, and then decrease and level off (Fig. 3B-B'). However, if friction is neglected in the model, the behavior is not quite recapitulated, with T_{VP} falling below T_{AP} after both tensions peak. Interestingly, if moderate friction is included, the model behavior is in line with experiment (Fig. 3E). For very large friction, however, the tension curves are flattened and peaks are not observed. Therefore, comparison between the model and experimental tension versus time curves suggests that friction with the myoplasm is not fully negligible, but still the hydrodynamic length ℓ remains comparable or larger than R.

B. Myoplasm buckling model

The buckling model described in Section V depends on a handful of parameters, in addition to those given earlier: the myoplasm elastic modulus E; its Poisson ratio ν; the cytoplasm modulus E_{cyto}; the myoplasm thickness h; and the effective point of friction application a. To obtain the theoretical plots in Fig. 5E we have used the following values: $E = 1$ Pa ; $\nu = 0$; $E_{\mathrm{cyto}} = 1$ Pa; $h = 3$ μm; and $a = 100$ μm. To highlight the importance of the background cytoplasm elastic stiffness, $K = E_{\mathrm{cyto}}/R$ (up to a numerical factor), in Fig. 5E we have also considered smaller values: $E_{\mathrm{cyto}} = 10^{-2}$ Pa (dashed line), and $E_{\mathrm{cyto}} = 10^{-4}$ Pa (dotted line).

[1] J. S. Bois, F. Jülicher, and S. W. Grill, Phys Rev Lett **106**, 028103 (2011).

[2] A. C. Callan-Jones and R. Voituriez, New Journal of Physics **15**, 025022 (2013), URL `https://dx.doi.org/10.1088/1367-2630/15/2/025022`.

[3] P. Recho, T. Putelat, and L. Truskinovsky, Phys Rev Lett **111**, 108102 (2013).

[4] H. B. da Rocha, J. Bleyer, and H. Turlier, Journal of the Mechanics and Physics of Solids **164**, 104876 (2022).

[5] F. Prodon, P. Dru, F. Roegiers, and C. Sardet, J Cell Sci **118**, 2393 (2005).

[6] B. Audoly and Y. Pomeau, *Elasticity and geometry: from hair curls to the nonlinear response of shells* (Oxford University Press, 2010).

[7] L. D. Landau, L. P. Pitaevskii, E. M. Lifshitz, and A. M. Kosevich, *Theory of Elasticity* (Butterworth-Heinemann, 1986), 3rd ed., ISBN 075062633X, URL `http://www.amazon.com/Theory-Elasticity-Third-Theoretical-Physics/dp/075062633X/ref=sr_1_16?ie=UTF8&s=books&qid=1280929419&sr=8-16`.
