# Supplementary material for: Friction forces determine cytoplasmic reorganization and shape changes of ascidian oocytes upon fertilization
Source: Nat Phys. 2024 Jan 9;20(2):310–21. doi: 10.1038/s41567-023-02302-1 (PMC10866705; doi:10.1038/s41567-023-02302-1)
Supplement: Supplementary file 2 — Reporting Summary [file 41567_2023_2302_MOESM2_ESM.pdf]

nature portfolio

Corresponding author(s): *Double-anonymous peer review submissions: write DAPR and your manuscript number here instead of author names.*

Last updated by author(s): Oct 2, 2023

Reporting Summary

Nature Portfolio wishes to improve the reproducibility of the work that we publish. This form provides structure for consistency and transparency in reporting. For further information on Nature Portfolio policies, see our Editorial Policies and the Editorial Policy Checklist.

Statistics

For all statistical analyses, confirm that the following items are present in the figure legend, table legend, main text, or Methods section.

n/a	Confirmed	
☐	☒	The exact sample size (*n*) for each experimental group/condition, given as a discrete number and unit of measurement
☐	☒	A statement on whether measurements were taken from distinct samples or whether the same sample was measured repeatedly
☐	☒	The statistical test(s) used AND whether they are one- or two-sided<br>*Only common tests should be described solely by name; describe more complex techniques in the Methods section.*
☒	☐	A description of all covariates tested
☐	☒	A description of any assumptions or corrections, such as tests of normality and adjustment for multiple comparisons
☐	☒	A full description of the statistical parameters including central tendency (e.g. means) or other basic estimates (e.g. regression coefficient) AND variation (e.g. standard deviation) or associated estimates of uncertainty (e.g. confidence intervals)
☐	☒	For null hypothesis testing, the test statistic (e.g. *F*, *t*, *r*) with confidence intervals, effect sizes, degrees of freedom and *P* value noted<br>*Give P values as exact values whenever suitable.*
☒	☐	For Bayesian analysis, information on the choice of priors and Markov chain Monte Carlo settings
☒	☐	For hierarchical and complex designs, identification of the appropriate level for tests and full reporting of outcomes
☒	☐	Estimates of effect sizes (e.g. Cohen's *d*, Pearson's *r*), indicating how they were calculated

Our web collection on statistics for biologists contains articles on many of the points above.

Software and code

Policy information about availability of computer code

Data collection	No software was used for data collection
Data analysis	Fiji (NIH), Imaris 9.0, Ilastick, GraphPad Prism 8.0 and custom Python code.

For manuscripts utilizing custom algorithms or software that are central to the research but not yet described in published literature, software must be made available to editors and reviewers. We strongly encourage code deposition in a community repository (e.g. GitHub). See the Nature Portfolio guidelines for submitting code & software for further information.

Data

Policy information about availability of data

All manuscripts must include a data availability statement. This statement should provide the following information, where applicable:

- Accession codes, unique identifiers, or web links for publicly available datasets
- A description of any restrictions on data availability
- For clinical datasets or third party data, please ensure that the statement adheres to our policy

Source data for Figures 1B, C, E; 2B; 3B, C, D; 5B, B'; 6B, C, F; S1B; S3B, D; S6A; S7A, C'; S8D have been provided as Data_and_statistics supplementary file. All other data supporting the findings of this study are available from the corresponding author upon request.

Research involving human participants, their data, or biological material

Policy information about studies with human participants or human data. See also policy information about sex, gender (identity/presentation), and sexual orientation and race, ethnicity and racism.

Reporting on sex and gender	N/A
Reporting on race, ethnicity, or other socially relevant groupings	N/A
Population characteristics	N/A
Recruitment	N/A
Ethics oversight	N/A

Note that full information on the approval of the study protocol must also be provided in the manuscript.

Field-specific reporting

Please select the one below that is the best fit for your research. If you are not sure, read the appropriate sections before making your selection.

☒ Life sciences ☐ Behavioural & social sciences ☐ Ecological, evolutionary & environmental sciences

For a reference copy of the document with all sections, see nature.com/documents/nr-reporting-summary-flat.pdf

Life sciences study design

All studies must disclose on these points even when the disclosure is negative.

Sample size	No sample size calculation was performed. Sample sizes are different in each experiment and reported in the corresponding figure legend.<br>Sample size was chosen based on our experience and the work of others using oocytes as subject of study.<br>Cell 177, 1463-1479, 2019
Data exclusions	No data was excluded from the analysis
Replication	All attempts at replication were successful. At least 3 independent experiments were performed for the data shown, where an independent experiment was defined as oocytes extracted from different animals.
Randomization	No randomization method was used
Blinding	No blind allocation during data collection and analysis was performed

Reporting for specific materials, systems and methods

We require information from authors about some types of materials, experimental systems and methods used in many studies. Here, indicate whether each material, system or method listed is relevant to your study. If you are not sure if a list item applies to your research, read the appropriate section before selecting a response.

Materials & experimental systems

n/a	Involved in the study	
☐	☒	Antibodies
☒	☐	Eukaryotic cell lines
☒	☐	Palaeontology and archaeology
☐	☒	Animals and other organisms
☒	☐	Clinical data
☒	☐	Dual use research of concern
☒	☐	Plants

Methods

n/a	Involved in the study	
☒	☐	ChIP-seq
☒	☐	Flow cytometry
☒	☐	MRI-based neuroimaging

Antibodies

Antibodies used	Mouse anti-neurofilament 160 monclonal antibody, Sigma-Aldrich, N5264; Rabbit anti- phospho S6 ribosomal protein (Ser235/236) polyclonal antibody , Cell Signaling Technology, 2211; Rabbit anti-phospho Myosin light chain 2, Cell Signaling Technology, 3674S;

	Alexa Fluor 488 goat anti-mouse IgG (H+L), Molecular Probes, A-11029; Alexa Fluor 546 goat anti-rabbit IgG (H+L), Molecular Probes, A-11035 and Alexa Fluor 647 goat anti-rabbit IgG (H+L), Molecular Probes, A-21245
Validation	The anti-neurofilament antibody was validated through immunostaining in ascidian eggs and embryos (Chenevert et al., Plos One, 8, e52996, 2013; Sardet et al., Vertebrate Embryogenesis: Methods in Molecular Biology 770, 365-400, 2011). The anti-phospho S6 ribosomal protein antibody was validated through immunostaining in ascidian eggs and embryos (Paix et al., RNA Detection and Visualization: Methods in Molecular Biology 714, 49-70, 2011). The anti-phospho Myosin light chain antibody was validated through immunostaining in ascidian embryos (Sherrard et al., Current Biology 20, 1499-1510, 2010).

Animals and other research organisms

Policy information about studies involving animals; ARRIVE guidelines recommended for reporting animal research, and Sex and Gender in Research

Laboratory animals	No laboratory animals were used
Wild animals	Adult Phallusia mammillata were collected by underwater divers in the Baie de Morlaix (Roscoff, France) from the Atlantic Ocean and sent to the Institute of Science and Technology Austria (Austria) within 24h of collection. Once in our laboratory they were kept in a salt water aquarium until they were dissected for eggs and sperm collection
Reporting on sex	The sex of the embryos is unknown
Field-collected samples	No filed-collected samples were used
Ethics oversight	As invertebrate organisms, no ethical permission was required

Note that full information on the approval of the study protocol must also be provided in the manuscript.
